# The elbow: review of anatomy and common collateral ligament complex pathology using MRI

**DOI:** 10.1186/s13244-019-0725-7

**Published:** 2019-04-03

**Authors:** José Acosta Batlle, Luis Cerezal, María Dolores López Parra, Beatriz Alba, Santiago Resano, Javier Blázquez Sánchez

**Affiliations:** 1 Radiology Department, Hospital Universitario Ramón y Cajal, Carretera de Colmenar Viejo Km. 9,100, 28034 Madrid, Spain; 2 Radiology Department, DMC-Diagnóstico Médico Cantabria, Castilla 6-Bajo, 39002 Santander, Spain

**Keywords:** MR imaging of the elbow, Elbow anatomy, Elbow instability, Imaging technique, Ligament injuries

## Abstract

The elbow is a complex joint whose stability is imparted by osseous and soft-tissue constraints. Anatomical and biomechanical knowledge of the supporting structures that provide stability to the medial and lateral elbow is essential to correctly interpret the pathological findings. Conventional MRI and MR arthrography are the imaging modalities of choice in the evaluation of elbow ligament injuries. Elbow instability can be classified according to timing (acute, chronic, or recurrent), the direction of displacement, the degree of displacement, and the articulations involved. This article reviews the MR imaging protocols recommended for each diagnosis and the normal anatomy and biomechanical aspects of the medial and lateral collateral ligament complex. We also present multiple cases of typical and atypical patterns of injury.

## Key points

- An accurate imaging diagnosis contributes to the management of acute and chronic injuries of the elbow.
- The anterior band of the medial or ulnar collateral ligament complex is the main stabilizer against valgus and internal rotation stress.
- The lateral collateral ligament complex resists excessive varus and external rotational stress. The lateral ulnar collateral ligament is the most important in terms of stability.
- Conventional MRI and MR arthrography are the imaging modalities of choice in the evaluation of elbow ligament injuries.
- Posterolateral rotatory instability is the most common pattern of recurrent elbow instability.

## Introduction

The elbow is a complex joint whose stability is imparted by osseous as well as soft-tissue constraints, and injuries often involve several of these structures. The anterior band of the ulnar or medial collateral ligament (MCL) complex is the main static stabilizer of the elbow against valgus and internal rotation stress. The lateral collateral ligament (LCL) complex resists excessive varus and external rotational stress. The lateral ulnar collateral ligament is the most important in terms of stability. The classification of elbow dislocations is based on the direction of dislocation: posterior, posterolateral, posteromedial, lateral, medial, or divergent. Elbow dislocation is also classified as simple, without associated fracture, or complex, with an associated fracture. The radial head is usually fractured in adults with a complex elbow dislocation [1]. The superior soft-tissue contrast of the magnetic resonance imaging (MRI) provides simultaneous evaluation of bone, hyaline cartilage, and soft tissue, allowing for assessment of all the static and dynamic stabilizers thus making accurate diagnoses possible with a single examination. In this article, we review the MRI protocols recommended for each diagnosis and the normal anatomy and biomechanical aspects of the MCL complex, the LCL complex, and the joint capsule. A better understanding of their anatomy and their relationship with adjacent structures is necessary to improve the detection of abnormalities. We also present multiple cases of typical and atypical patterns of injury of the MCL and LCL complex.

## Imaging technique

MRI of the elbow is best performed on a high-field strength magnet. To obtain adequate images of the ligamentous structures in the elbow, it is essential to use surface coils [2]. Circumferential and phased array coils improve signal to noise and are therefore preferable. A wrist coil can be used in small adults and children when a large field of view is not needed. Larger patients can be imaged with a flexible coil, anterior neck coil, shoulder coil, or knee coil. A larger coil is especially useful when the patient cannot fully extend the elbow or when the patient needs to be imaged in the prone position with the arm overhead [3].

Placing the elbow at the isocenter of the scanner, where magnetic field homogeneity and gradient uniformity are best, usually requires prone positioning with the arm of interest extended overhead (“superman” position). This position can be uncomfortable and therefore prone to motion artifact, although it can be improved by adding motion-insensitive sequences, such a propeller. Modern phased-array multichannel coils allow placement of the elbow by the side of the patient in a supine position, which is more comfortable and less prone to motion. Nevertheless, achieving high-quality imaging with fat suppression can be difficult. The use of manual shimming and manual prescan can often correct this problem. The use of short-tau inversion recovery for fat suppression or methods of fat/water separation can also be useful [4, 5].

**Fig. 1:**
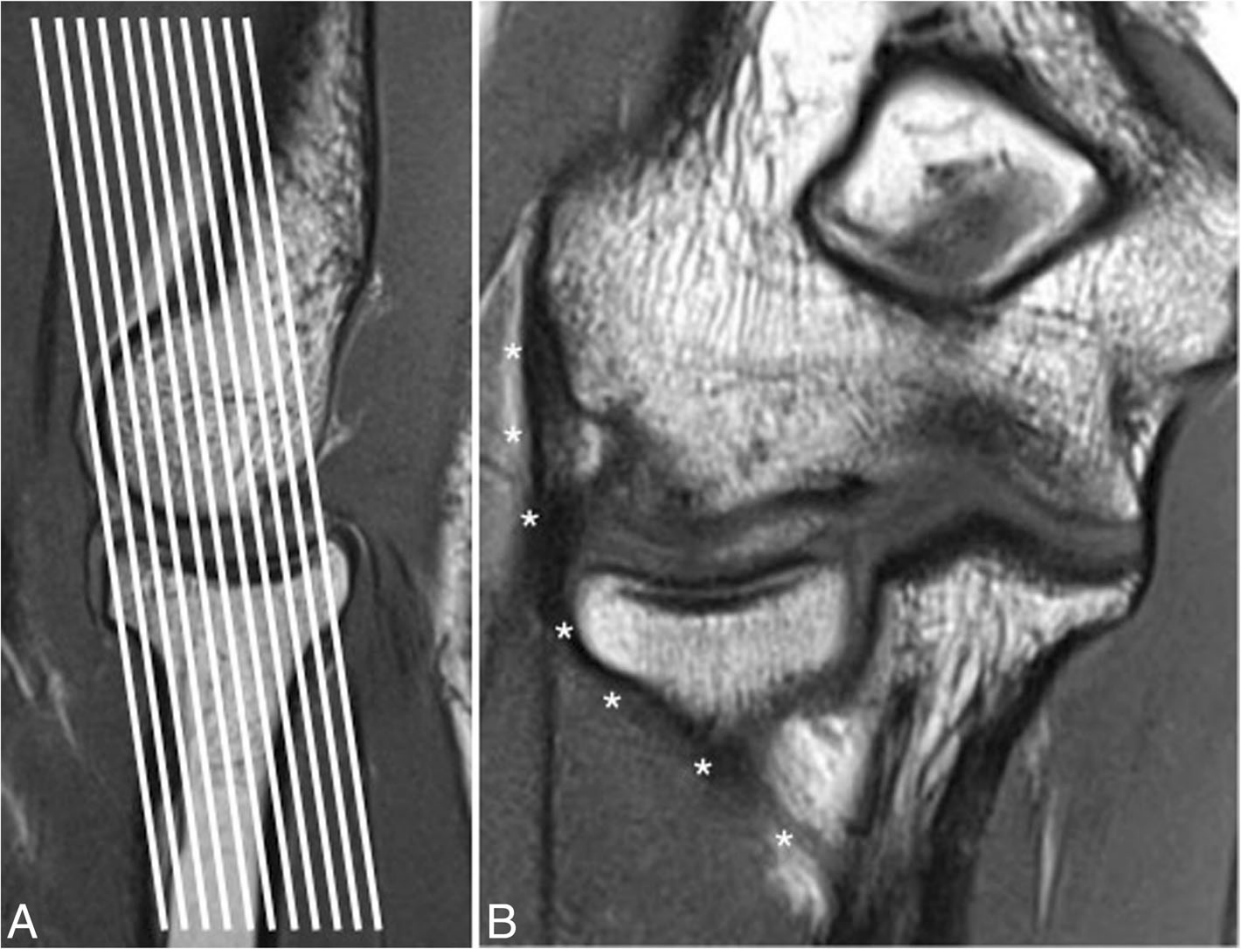
Modified oblique coronal plane of the elbow used to optimally visualize the collateral ligaments. **a** Sagittal T1-weighted MRI showing the modified oblique coronal plane (multiple white lines) oriented 20–30° posterior to the long axis of the humeral diaphysis. **b** Coronal T1-weighted MRI showing the intact lateral ulnar collateral ligament (white asterisks)

At our hospital, the routine elbow protocol consists of coronal T1-weighted (w), coronal T2-w FS, axial T2-w FS, axial T1-w, axial intermediate-w FS, sagittal T1-w FS, and sagittal T2-w fast spin-echo sequences (FSE). All sequences are performed with a 12 to 14 cm field of view and a 256 × 192 or 256 × 256 pixels matrix. Axial, sagittal, and coronal images of the elbow are acquired with a 2.5-mm slice thickness and a 0.5-mm section spacing [5]. The collateral ligaments of the elbow are optimally visualized in a 20° posterior oblique coronal plane in relation to the humeral diaphysis with the elbow extended (Fig. 1) and a coronal plane aligned with the humeral diaphysis with the elbow flexed 20 to 30°. These modified coronal planes are obtained using a sagittal scout image [6, 7]. However, correlation with the axial and sagittal images is often advisable to confirm suspected pathology. The normal ligaments appear as homogeneously hypointense structures relative to adjacent skeletal muscle, since they are primarily composed of type I collagen fibers [1, 7].

**Fig. 2:**
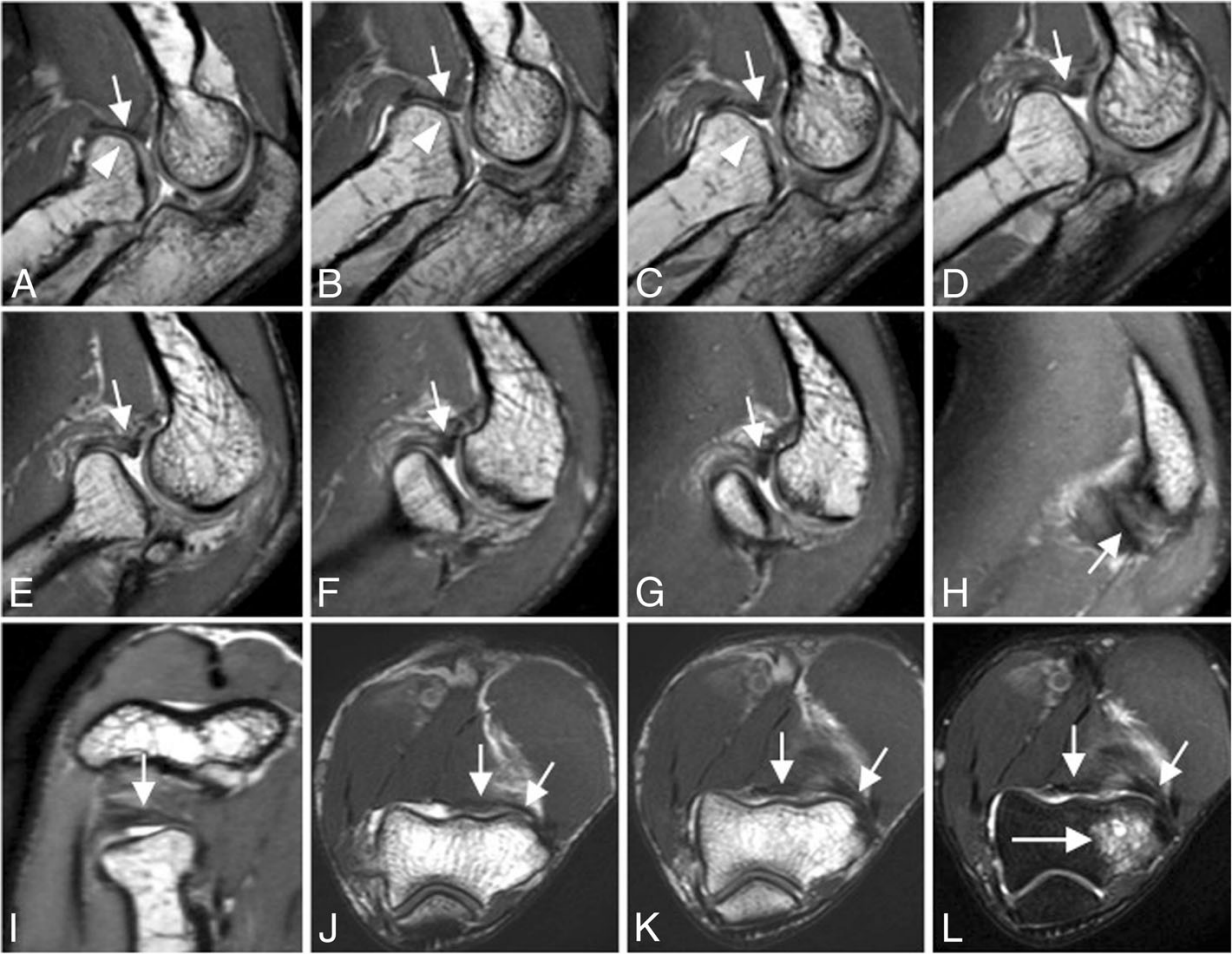
A 46-year-old man with recurrent painful click in the elbow. Consecutive sagittal 3D-FSE PD-weighted MRI (**a** to **h**), consecutive axial 3D-FSE PD-weighted (**i** to **k**), and axial FS PD-weighted MRI (**l**) show superior displacement of annular ligament interposing between the radial head and the capitellum (white short arrows). Radial head deformity (white arrowheads). Capitellar osteochondral lesion (long white arrow)

The introduction of an isotropic three-dimensional (3D) imaging technique (Fig. 2) has shown promise in the visualization of anatomy and pathology of the elbow, as well as in cartilage quantification. Isotropic imaging eliminates slice gaps and reduces partial volume artifact. The use of isotropic voxels allows images to be reformatted retrospectively into arbitrary planes to better visualize oblique fibers of some ligaments: a significant decrease in scan time results as reformats can only be manipulated from one acquisition. Furthermore, 3D-FSE scans can be limited by blurring, although extended echo trains are making this technique more feasible [4].

**Fig. 3:**
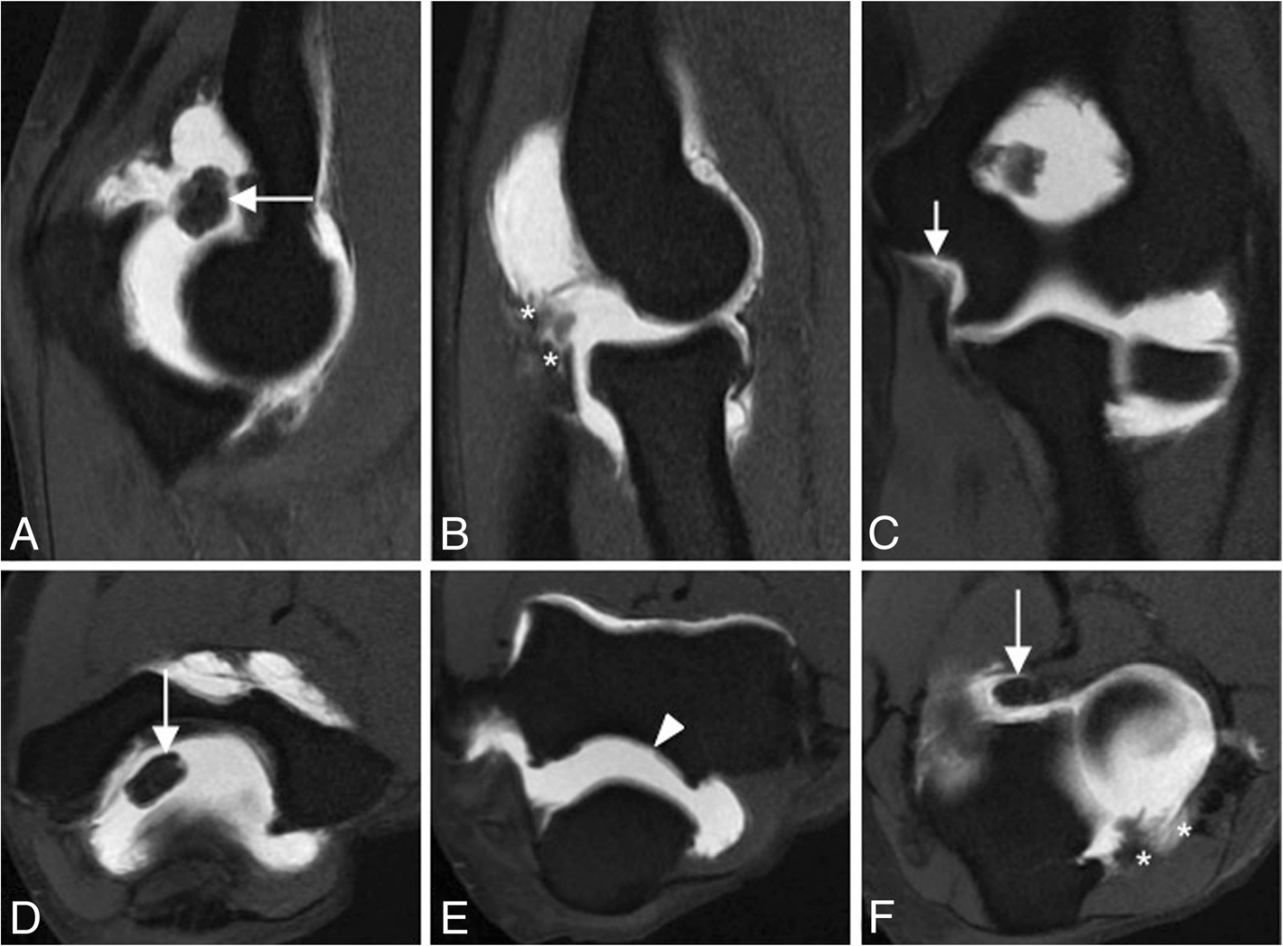
Sagittal FS T1-weighted direct MR arthrographic images (**a**, **b**), coronal FS T1-weighted direct MR arthrographic image (**c**), and axial FS T1-weighted direct MR arthrographic images (**d**–**f**) showing a proximal partial-thickness proximal tear of the anterior bundle of the medial collateral ligament complex (short arrow), disruption and stripping of the posterior and lateral capsular structures (white asterisks), intra-articular bodies (long arrows), and full-thickness defect at the posterior joint line (arrowhead). Note ulnohumeral incongruity (**a**) and radiocapitellar incongruity (**b**)

Magnetic resonance (MR) arthrography of the elbow is very helpful to evaluate intra-articular loose bodies, osteochondral and chondral lesions, capsular and ligamentous injury, medial elbow pain in the throwing athlete, and elbow dislocations [8] (Fig. 3).

Direct MR arthrography distends the joint compartment, allowing for better delineation and visualization between tissues. It also allows detection of abnormal communication between joint compartment and extra-articular soft tissues. Indirect MR arthrography is less invasive and may be useful in some cases when direct MR arthrography is not feasible [9].

When direct arthrography is performed, the joint capsule can be injected with a mixture of gadolinium, saline or ropivacaine, and iodinated contrast material. With fluoroscopic guidance, the joint can be entered laterally over the radial head. Alternatively, a posterior approach has been suggested to avoid the radial collateral ligament complex or a posterolateral approach to also avoid the triceps tendon [5, 8, 10]. Then, 6–10 mL is normally sufficient to adequately distend the joint. The imaging protocol consists of fat suppressed T1-w fast spin-echo sequences in the axial, coronal, and sagittal planes. It should also include fat suppressed T2-w fast spin-echo images or STIR images in at least one plane in order to detect osseous and other extra-articular pathologies. Gradient-echo sequences or 3D volumetric sequences are also very useful. Some authors have proposed using saline solution when there is a documented allergy to gadolinium-based compounds. When performing MR arthrography with intra-articular saline solution, fat-suppressed T2-w sequences are essential and should replace the fat-suppressed T1-w sequences in the standard direct MR arthrographic protocol [8].

## Normal anatomy

The elbow joint consists of three different articulations within a single synovial capsule: the ulnohumeral, the radiocapitellar, and the radioulnar joints. The first two joints function as a hinge, permitting flexion and extension; the last two joints accomplish the pivot motion of pronation and supination, and are functionally linked to the distal radioulnar joint and the wrist. The physiologic range of motion is 0 to 140° for flexion-extension movements and 0 to 180° for supination-pronation movements [5, 11].

The stability of the elbow joint depends on the integrity of several osseous and soft-tissue structures. The elbow has both static and dynamic constraints. The three primary static stabilizing structures are the ulnohumeral joint, which provides about 33% of valgus stability; the anterior bundle of the medial or ulnar collateral ligament complex, which provides about 54% of valgus stability; and the lateral ulnar collateral ligament component of the radial or lateral collateral ligament complex. The secondary static stabilizing structures include the radiocapitellar joint, the common flexor and extensor origins, and the joint capsule [1, 12]. The most important static soft-tissue constraints are the lateral ulnar collateral ligament and the anterior bundle of the medial collateral ligament [5, 7, 13]. The ulnohumeral joint is the most important osseous stabilizer of the elbow, providing primary stability below 20° or above 120° of flexion [5, 14].

Dynamic stabilizers include the muscles that cross the elbow joint and produce compressive forces at the articulation, being the anconeus, triceps, and brachialis muscles the most important [6, 15].

The capsule of the elbow is reinforced by the collateral ligaments on the lateral and the medial side of the joint, but it is relatively weak anteriorly and posteriorly.

### Medial or ulnar collateral ligament complex

**Fig. 4:**
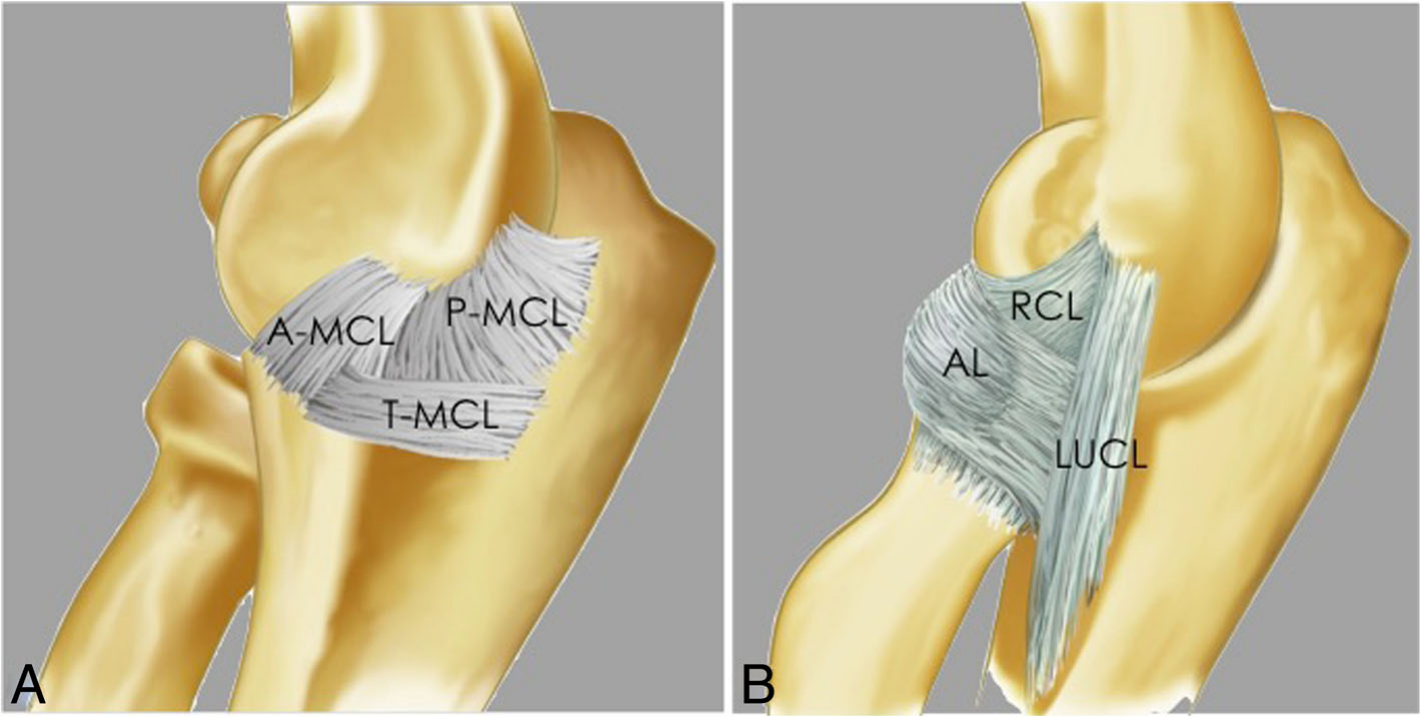
Elbow illustration. Medial view (**a**) demonstrating the medial collateral ligament complex: anterior bundle of the medial collateral ligament (A-MCL), posterior bundle of the medial collateral ligament (P-MCL), and transverse ligament (T-MCL). Lateral view (**b**) demonstrating the lateral collateral ligament complex: lateral ulnar collateral ligament (LUCL), radial collateral ligament (RCL), and annular ligament (AL)

On the medial side of the elbow joint, the MCL complex is comprised of three bundles: the anterior, posterior, and transverse bundles (Fig. 4a). Twenty-three percent of people have an accessory ulnar collateral ligament, which originates on the posterior joint capsule and inserts onto the transverse bundle [16, 17].

**Fig. 5:**
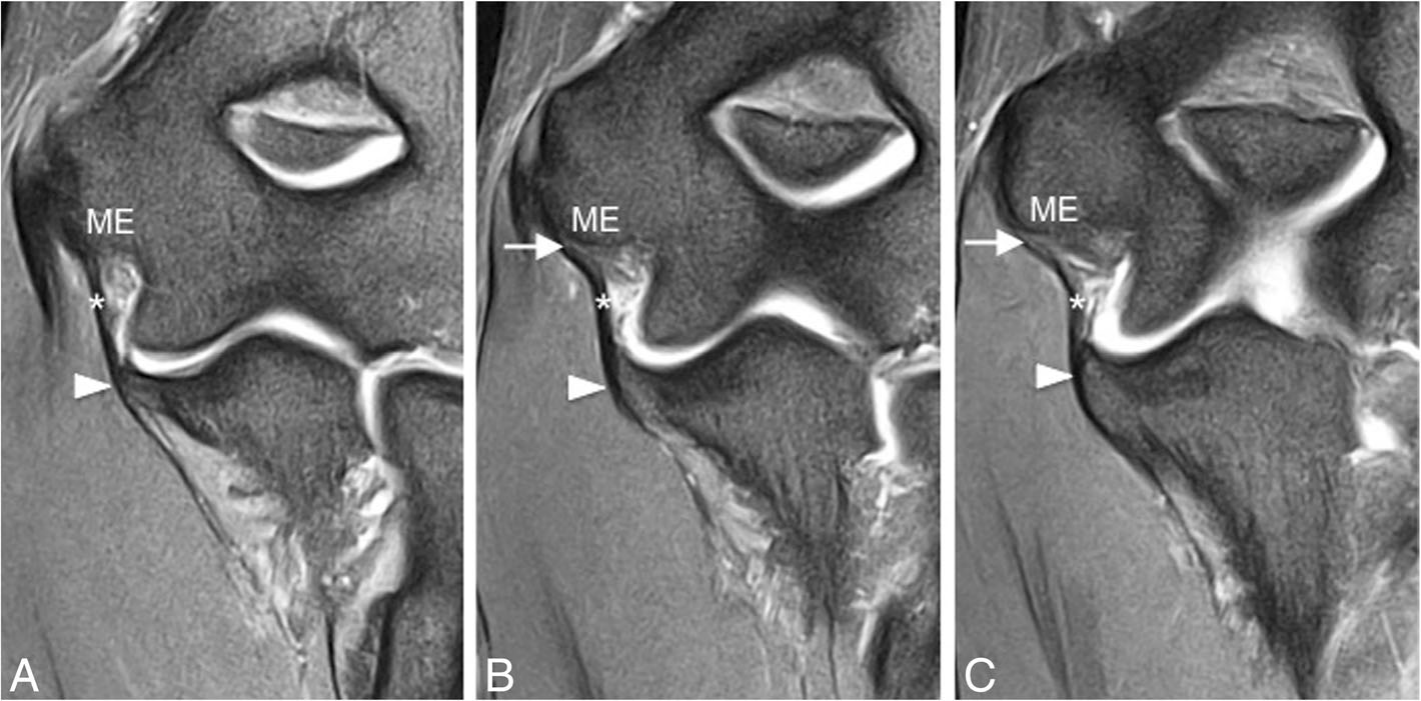
Consecutive coronal FS PD-weighted MRI (**a**–**c**) showing the intact anterior bundle of the medial collateral ligament (A-MCL) complex (white asterisks). It extends from the inferior aspect of the medial epicondyle (ME) to the sublime tubercle of the ulna (white arrowheads). Note the typical striated appearance of the normal proximal A-MCL (white arrows)

**Fig. 6:**
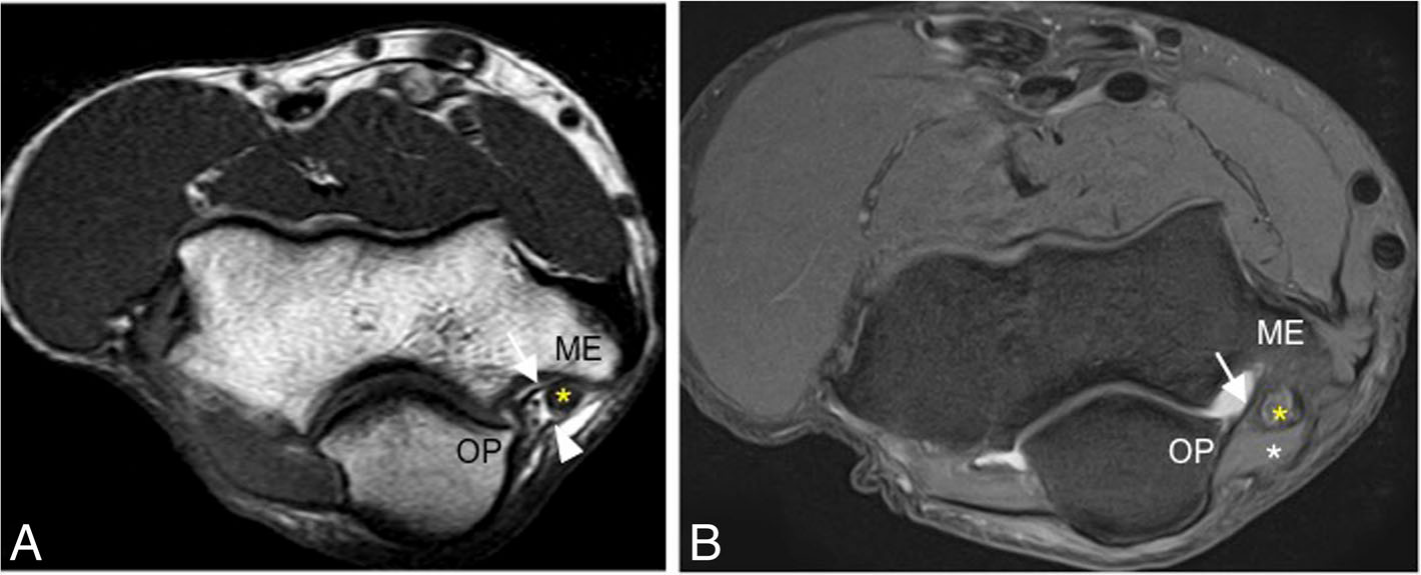
Axial T1-weighted MRI (**a**) and axial FS PD-weighted MRI (**b**) in two different healthy volunteers showing the intact posterior bundle of the medial collateral ligament complex (white arrows). It extends from the posterior aspect of the medial epicondyle (ME) to the medial aspect of the olecranon process (OP). Cubital tunnel retinaculum (white arrowhead). Ulnar nerve (yellow asterisks). Anconeus epitrochlearis muscle (white asterisk)

The anterior bundle (A-MCL) arises from the inferior margin of the medial epicondyle and inserts at the sublime tubercle of the ulnar coronoid process (Fig. 5). The A-MCL is composed of a superficial and a deep layer [18]**.** The superficial layer is a separate structure from the joint capsule and is considered to be associated with deep fibers of the flexor digitorum superficialis tendon [14]. The A-MCL can be separated into two bands, which are taut during different degrees of flexion/extension [19]. The anterior band of the anterior bundle is the most important static stabilizer of the elbow against valgus and internal rotation [20]. The posterior bundle (P-MCL) originates at the posterior aspect of the medial epicondyle of the humerus and attaches to the medial aspect of the olecranon process, forming the floor of the cubital tunnel (Fig. 6). During normal elbow flexion, the flexor carpi ulnaris aponeurosis tenses while the medial collateral ligament relaxes and bulges superficially. These changes result in decreased volume and increased pressure inside the cubital tunnel during flexion. The transverse bundle originates on the proximal medial olecranon and runs distally to insert just distal to the coronoid. Because this ligament originates and inserts on the ulna, it does not provide significant stability [17]. In addition, the ligament is variably present. This combination makes the ligament of relatively low clinical and radiological importance***.*** On MR imaging, the proximal A-MCL has a striated appearance in 87–90% of healthy volunteers, which should not be confused with injury (Fig. 5) [5, 14, 17, 21]. The A-MCL is best visualized on coronal and axial slices. The P-MCL can be traced from origin to insertion on the coronal images and identified as the floor of the cubital tunnel on axial slices [22].

### Lateral collateral ligament complex

**Fig. 7:**
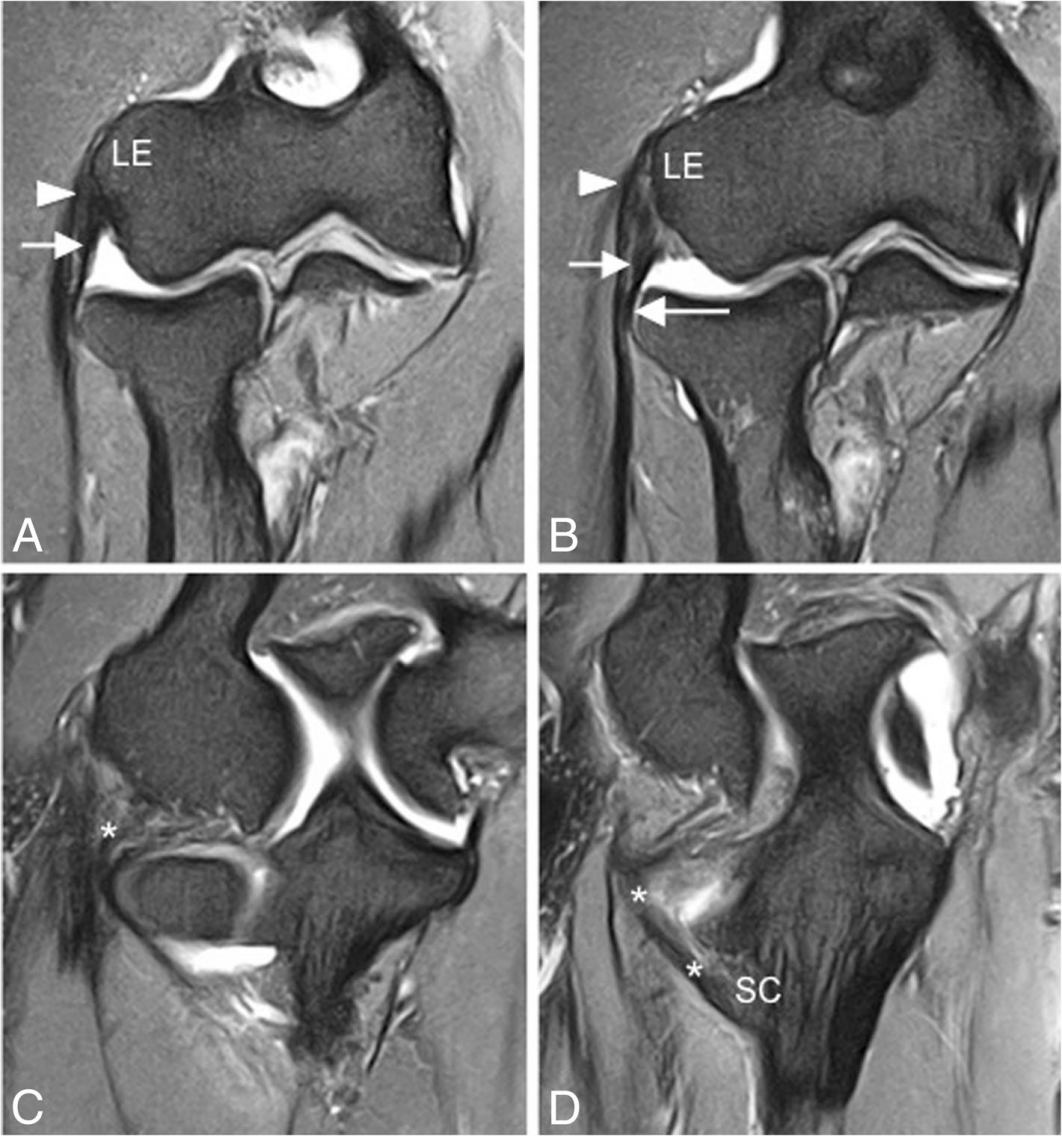
Consecutive coronal FS PD-weighted MRI (**a**–**d**) showing the intact lateral ulnar collateral ligament (white asterisks) and radial collateral ligament (short arrows). The lateral ulnar collateral ligament wraps around the posterior aspect of the radial neck. Annular ligament (long arrow). Common extensor tendon (white arrowheads). Lateral epicondyle (LE). Supinator crest of the ulna (SC)

**Fig. 8:**
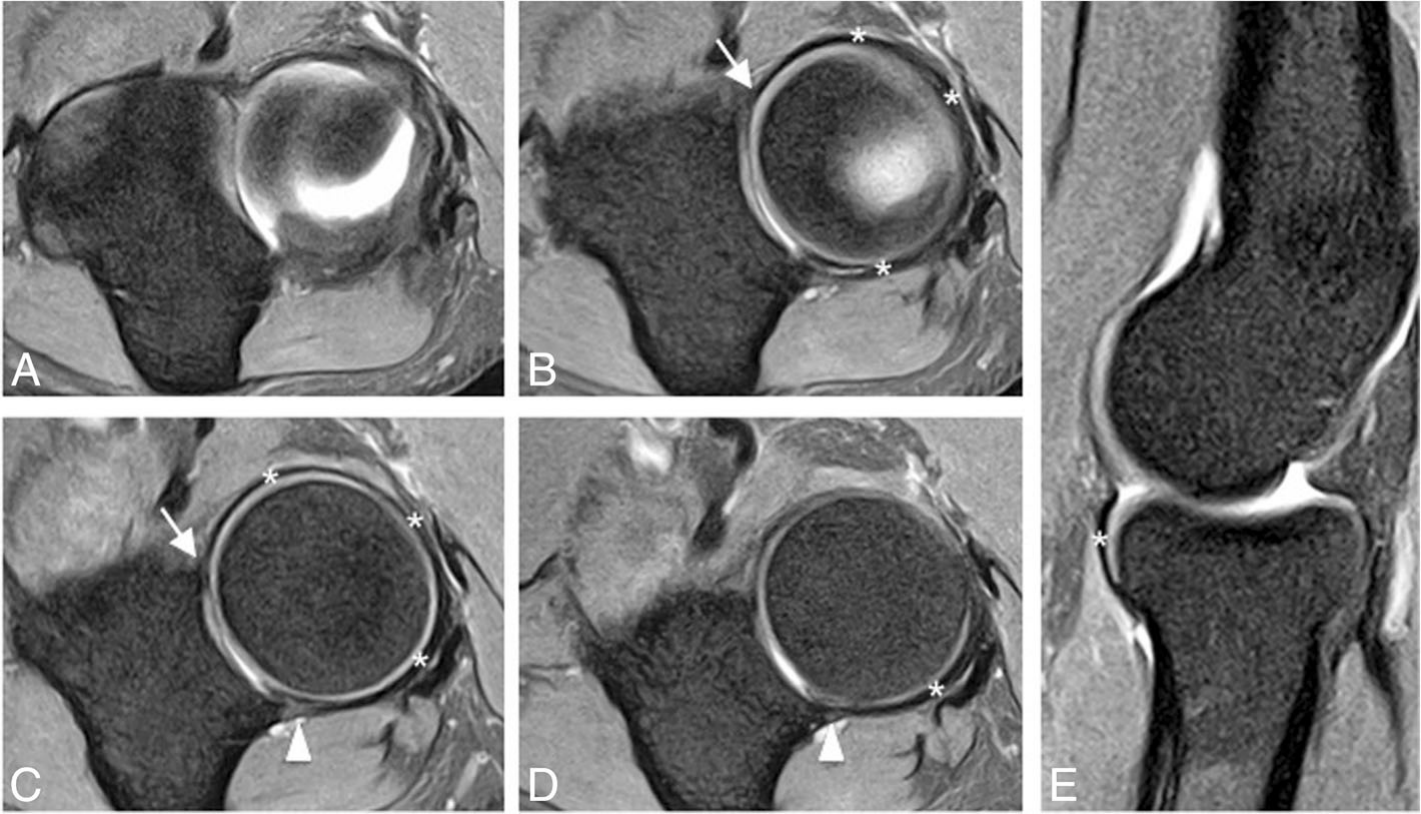
Axial FS PD-weighted MRI (**a**–**d**) and sagittal FS PD-weighted MRI (**e**) showing the intact annular ligament (white asterisks). Anterior attachment of the annular ligament (white arrow). The posterior attachment of the annular ligament can be fenestrated (white arrowheads). The annular ligament encircles the periphery of the radial head (white asterisks in **b-d**)

On the lateral side of the elbow joint, the LCL complex is comprised of three primary structures: the radial collateral ligament proper (RCL), the annular ligament (AL), and the lateral ulnar collateral ligament (LUCL) (Fig. 4b). One-third of individuals have an accessory lateral collateral ligament, which runs from the annular ligament to the supinator crest of the ulna [16, 17]. The RCL is a fan-shaped ligament that originates at the lateral epicondyle of the humerus and runs longitudinally underneath the common extensor tendon blending with the anterior annular ligament (Fig. 7). The RCL is best seen on coronal images [16]. The AL wraps around the anterior radial head, originating and inserting on the ulnar sigmoid notch, and is best seen on axial and sagittal images [16, 23] (Fig. 8). The anterior attachment of the AL to the ulna is observed as a single band, but the posterior attachment can be fenestrated normally [24] (Fig. 8c, d). This thick band primarily serves to stabilize the proximal radioulnar joint. The LUCL also originates at the lateral epicondyle of the humerus and partially blends in with the AL as it travels distally to insert on the supinator crest of the ulna [16, 17, 23] (Fig. 7). On MRI, it is incompletely visualized in up to 23% and it has a striated appearance in 78% of healthy volunteers [21]. The LUCL can be seen on coronal and sagittal images (16). In anatomic dissections, the humeral attachment of the LUCL is indistinguishable from that of the RCL because they both originate from the inferior aspect of the lateral epicondyle [25, 26]. The LUCL is considered to be the primary stabilizer of the elbow joint against posterolateral rotatory instability. It prevents the ulna from rotating around its long axis away from the trochlea [27, 28]. The LUCL stabilizes all three articulations of the elbow and contributes to the resistance of the rotatory forces of varus and external rotation. The common extensor tendon origin is the secondary soft-tissue stabilizer of the lateral elbow with a stronger contribution to stability during pronation of the forearm [29].

## Ligamentous pathology

Ligament injuries can be classified into three grades. Grade I sprain: MR imaging shows increased signal intensity within the ligament on T1- and T2-w images. Partial-thickness tear or grade II sprain: MR imaging demonstrates focal partial discontinuity of ligament fibers with hyperintense fluid signal extending partially through the ligament, often associated with swelling of the ligament. Full-thickness tear or grade III sprain: MR imaging shows complete disruption of the ligament with a fluid gap between the torn ligament fibers, and extra-capsular extravasation of joint fluid [1].

**Fig. 9:**
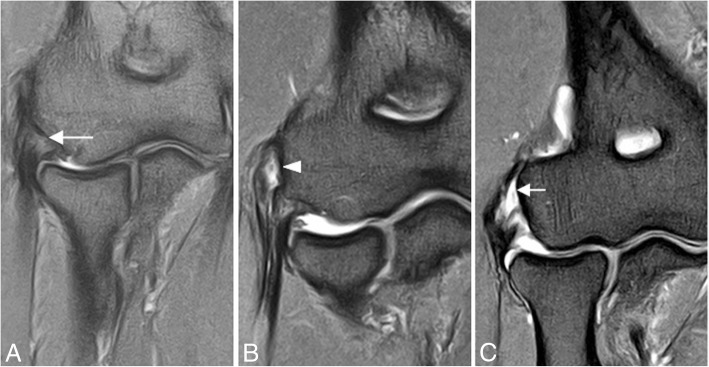
Three different patients with lateral elbow pain. Coronal FS PD-weighted MRI (**a**–**c**) showing common extensor tendinosis (long arrow), common extensor tendon partial-thickness tear (arrowhead), and common extensor tendon full-thickness tear (short arrow)

**Fig. 10:**
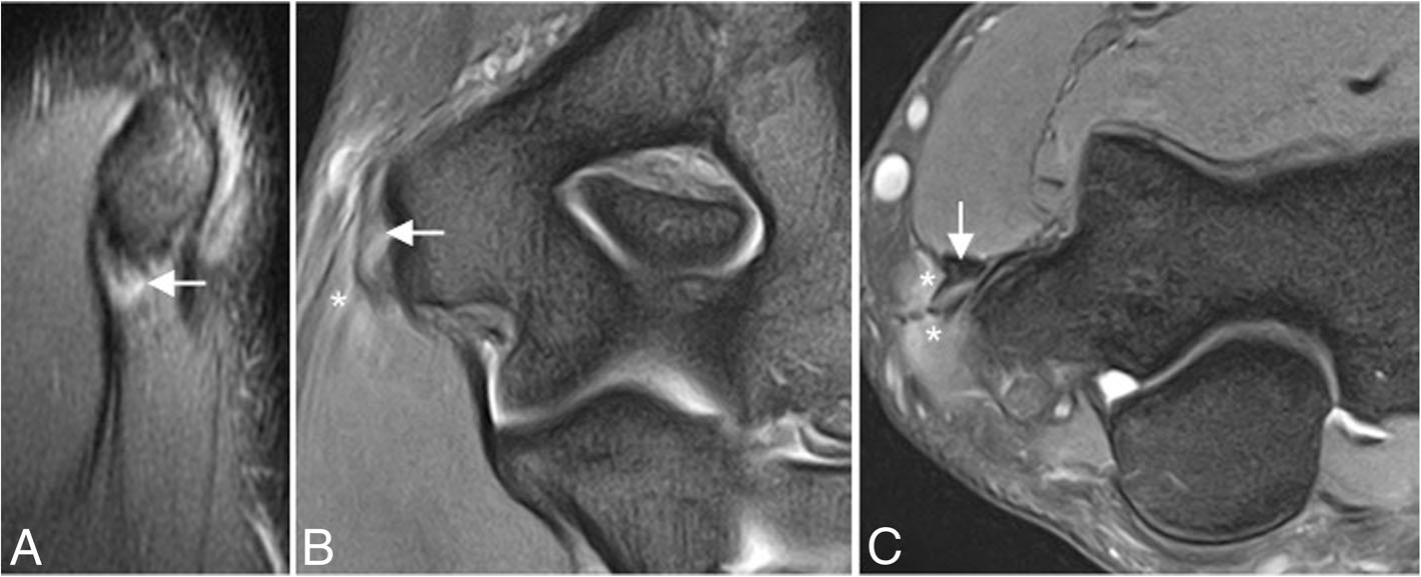
A 45-year-old man with medial elbow pain. Sagittal PD-weighted MRI (**a**), coronal FS PD-weighted MRI (**b**), and axial FS PD-weighted MRI (**c**) showing a common flexor tendinosis with intermediate signal within a diffusely thickened flexor tendon origin (white arrows). Note flexor digitorum superficialis and palmaris longus muscle edema (white asterisks)

**Fig. 11:**
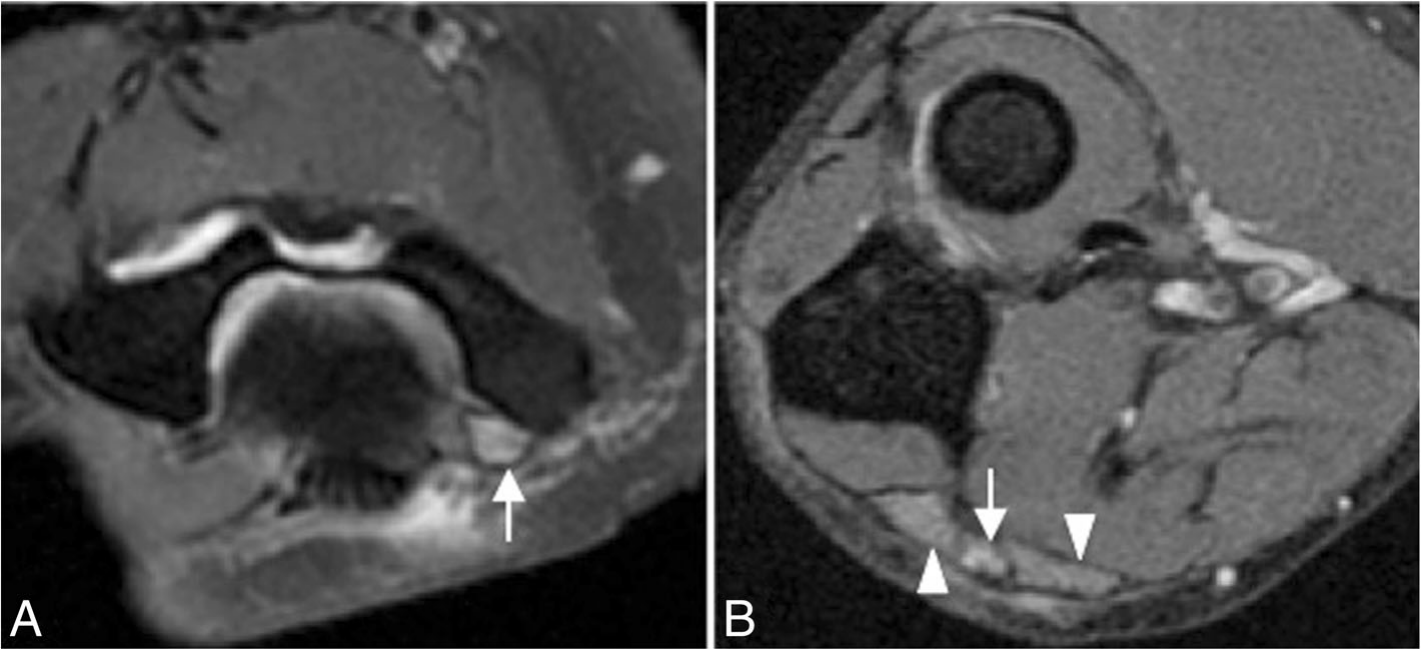
A 60-year-old woman with pain, muscle weakness, and paresthesias. Axial FS PD-weighted MRI (**a**, **b**) showing an enlargement and hyperintense ulnar nerve in the cubital tunnel (white arrow) and subacute denervation of the flexor carpi ulnaris muscle (white asterisks)

**Fig. 12:**
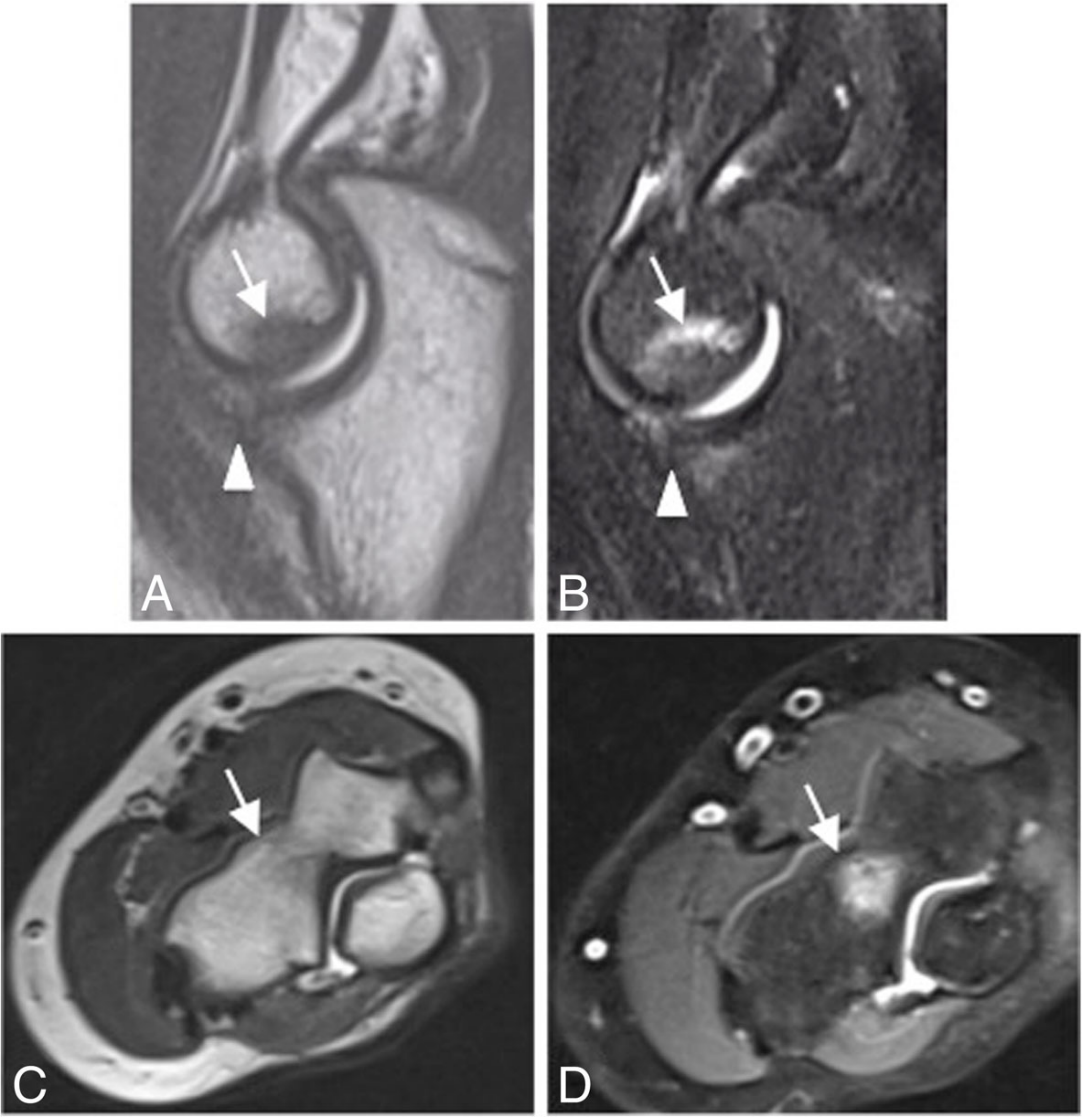
Sagittal T2-weighted MRI (**a**), sagittal FS PD-weighted MRI (**b**), axial T2-weighted MRI (**c**), and axial FS PD-weighted MRI (**d**) demonstrate an osteochondral lesion of the trochlea (white arrows), and a non-displaced fracture of the coronoid process (white arrowheads)

The differential diagnosis for elbow ligamentous injuries includes tendon pathology (Figs. 9 and 10), osteochondral impaction injuries to the radiocapitellar and ulnohumeral articular surfaces (Fig. 11), and nerve impingement or neuritis (Fig. 12) [1].

### Medial collateral ligament complex injury

The main function of the MCL complex is to maintain medial joint stability to valgus stress. The A-MCL is the most important component of the ligamentous complex acting as the primary medial stabilizer of the elbow from 30° to 120° of flexion [22]. The P-MCL becomes a secondary stabilizer of the elbow when the joint is flexed beyond 90° [11].

**Fig. 13:**
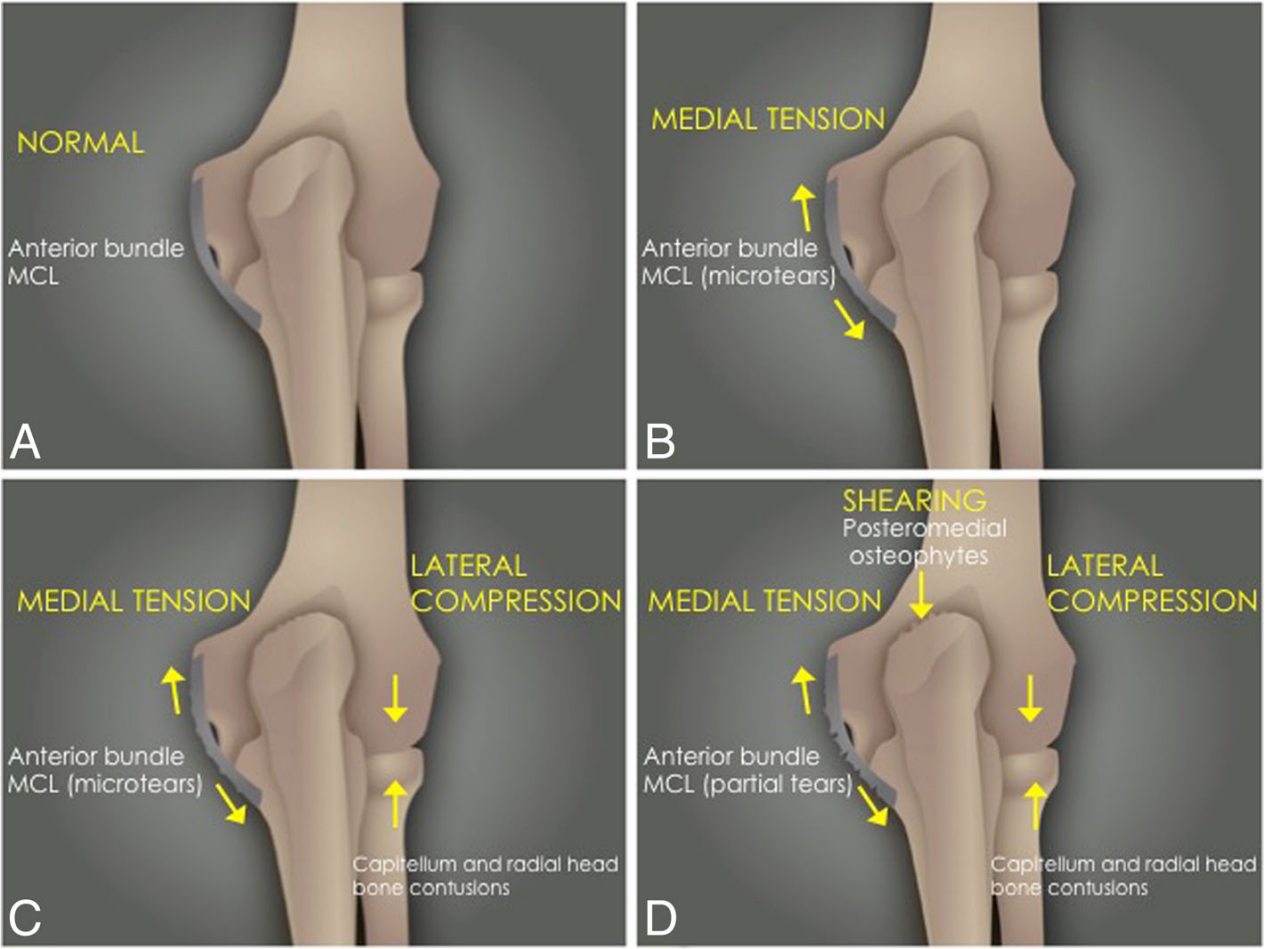
Posterior coronal view (**a**–**d**) of the elbow showing the mechanism of injury in the overhead athlete with repeated valgus stress

**Fig. 14:**
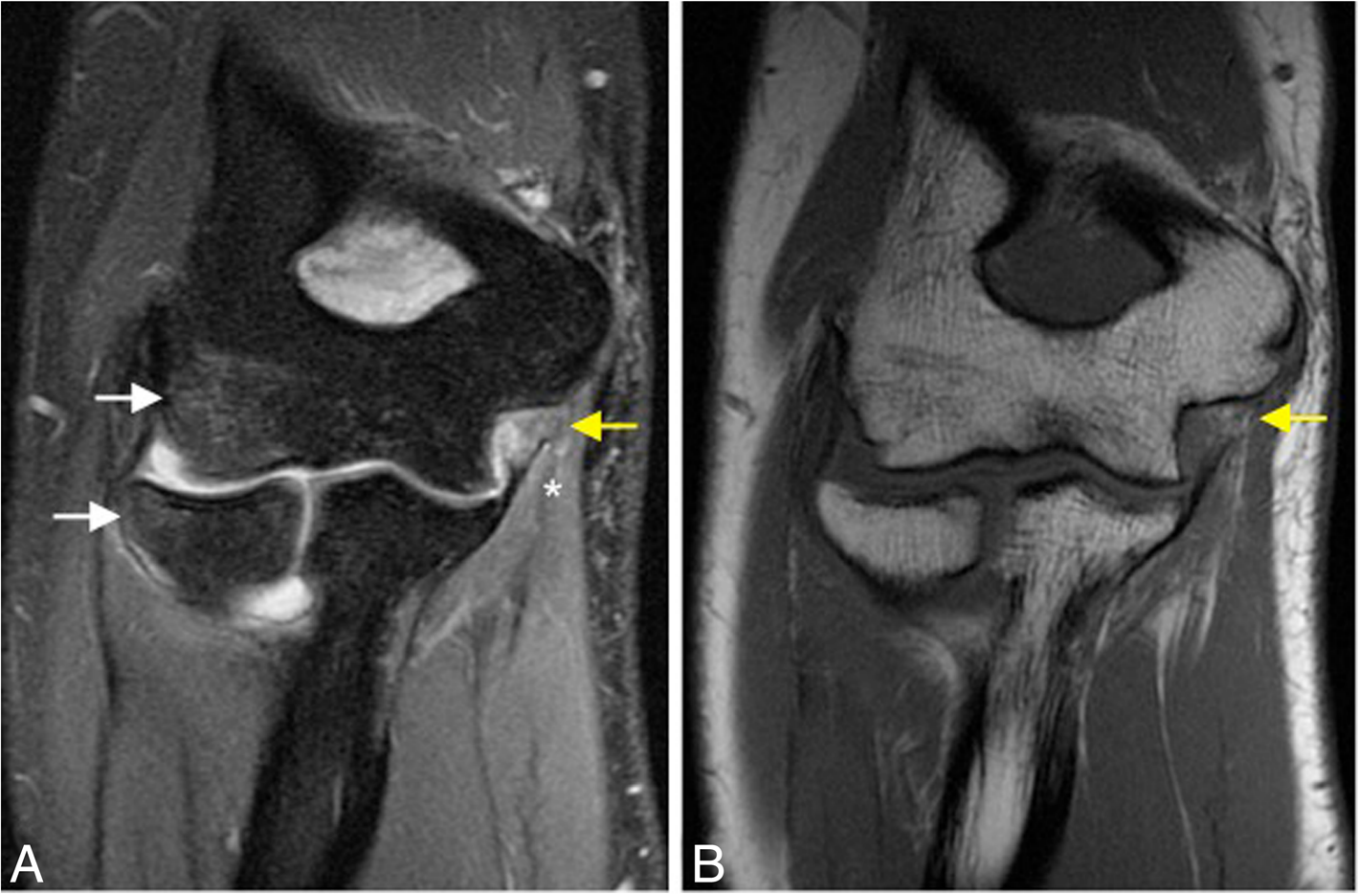
A 23-year-old man with pain and medial instability after a fall on the outstretched arm. Coronal FS PD-weighted MRI (**a**) and coronal T1-weighted MRI (**b**) showing an acute full-thickness proximal tear of the anterior bundle of the medial collateral ligament complex (yellow arrows), a flexor digitorum superficialis muscle edema (white asterisk), a radial head and posterior capitellar contusions (white arrows), and joint effusion

**Fig. 15:**
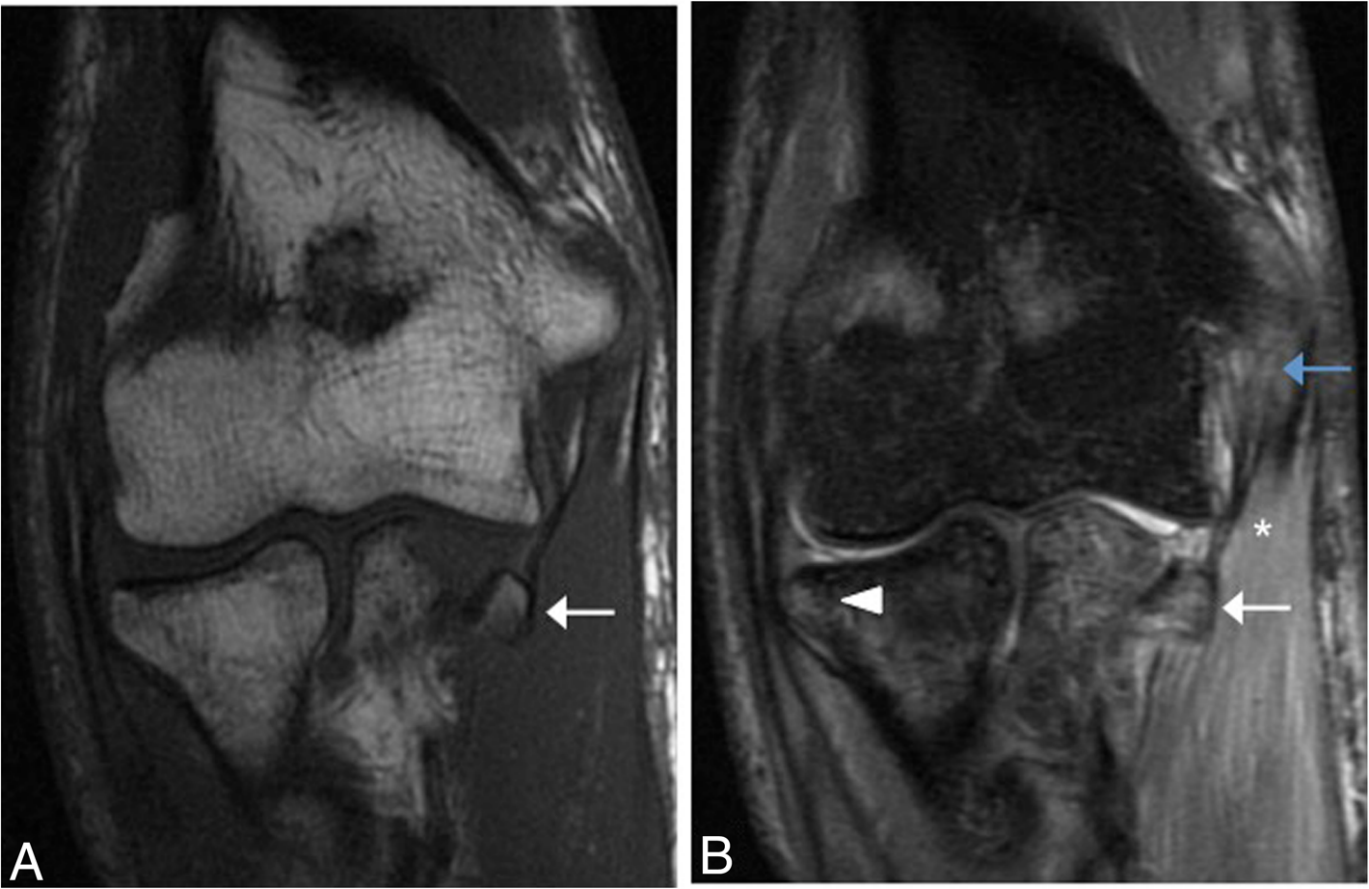
A 32-yer-old man with pain and medial instability after a fall on the outstretched arm. Coronal T1-weighted MRI (**a**) and coronal FS PD-weighted MRI (**b**) showing an acute avulsion fracture of the anterior bundle of the medial collateral ligament insertion in the sublime tubercle (white arrows), acute partial-thickness proximal tear of anterior bundle of the medial collateral ligament (blue arrow), flexor digitorum superficialis muscle edema (white asterisk), and radial head contusion (white arrowhead)

The most common mechanisms of MCL injury are chronic microtrauma from repetitive valgus stress, as seen in overhead and throwing athletes (baseball, javelin throwing, volleyball, golf, polo, and football), and after a fall on an outstretched hand. In the case of the latter, an acute tear of the MCL may be encountered. MCL rupture frequently occurs with posterior dislocation. Overhead throwing sports can result in medial elbow tension overload, lateral compression, and extension overload (Fig. 13) [1, 16]. The maximum stress on the MCL occurs during the late cocking and acceleration phases of throwing [7, 15]. Injury of the MCL is a common cause of medial elbow pain, and valgus instability in athletes. Repetitive insults to the ligament cause microscopic tears that progress to significant attenuation or frank tearing within its substance. Partial tears can be subtle and are well seen with magnetic resonance arthrography (Fig. 3c) [8, 9]. These patients may have associated findings, including lateral impaction and shearing of the articular surfaces of the capitellum and the radial head (Figs. 14 and 15). Reports have also described this process at the radial aspect of the trochlea.

**Fig. 16:**
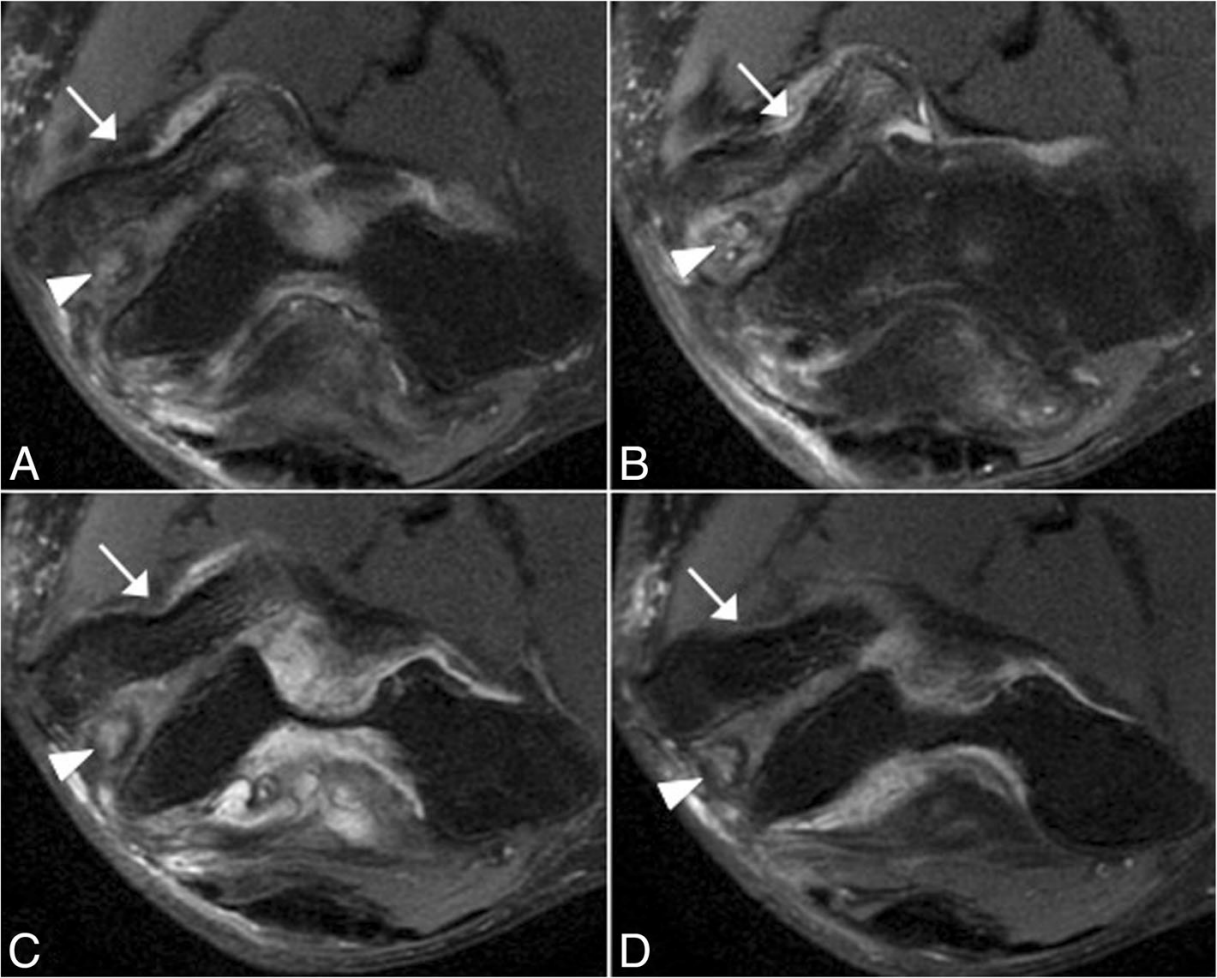
A 45-year-old woman with pain, muscle weakness, and paresthesias after a fall on an outstretched hand. Consecutive axial FS PD-weighted MRI (**a**–**d**) showing acute avulsion fracture of the medial epicondyle (white arrows). The ulnar nerve is trapped between osseous fragments (white arrowheads), showing intraneural increased signal intensity

**Fig. 17:**
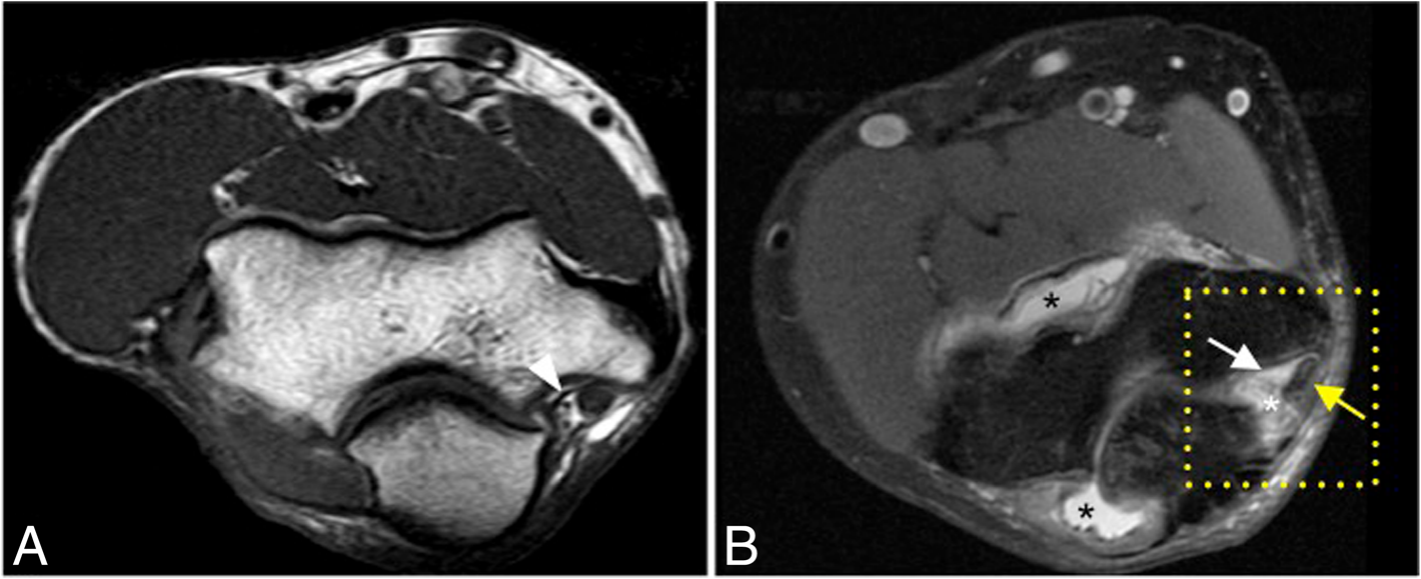
A 25-year-old man with pain and medial instability after a fall on an outstretched hand. Axial T1-weighted MRI (**a**) showing the intact right posterior bundle of the medial collateral ligament complex of a healthy volunteer (white arrowhead). Axial FS PD-weighted MRI (**b**) showing an acute tear of the left posterior bundle of the medial collateral ligament complex (white arrow), inflammatory changes in the soft tissue of cubital tunnel (white asterisk), displacement and flattening of the ulnar nerve (yellow arrow), and joint effusion (black asterisks)

In acute cases, MRI may show increased T2-w signal within the ligament, discontinuity of the ligament, and soft-tissue edema (Fig. 14). Midsubstance tears of the A-MCL are more common [1]. Distal (Fig. 15) and proximal avulsions (Fig. 16) are found less frequently [5, 7, 15]. Under surface partial tears in the distal insertion of the A-MCL complex have a characteristic aspect on MRI called the “T-sign,” which is produced by extension of fluid or contrast between the distal insertion of the ligament and the sublime tubercle. However, the distal insertion of the A-MCL complex can normally be up to 3 mm distal to the articular cartilage, especially in older patients, simulating the “T-sign” [5, 14]. The presence of other secondary signs of ligament injury such as ligament irregularity or periligamentous edema may be used to differentiate partial lesion from anatomical variant. The strain of the flexor digitorum superficialis frequently accompanies a MCL injury (Figs. 14 and 15). Thickening or acute disruption of the posterior bundle of the MCL may result in ulnar neuropathy [5, 22] (Fig. 17). Up to 40% of throwing athletes with MCL injuries and more than 50% with medial epicondylitis have ulnar neuropathy [22].

**Fig. 18:**
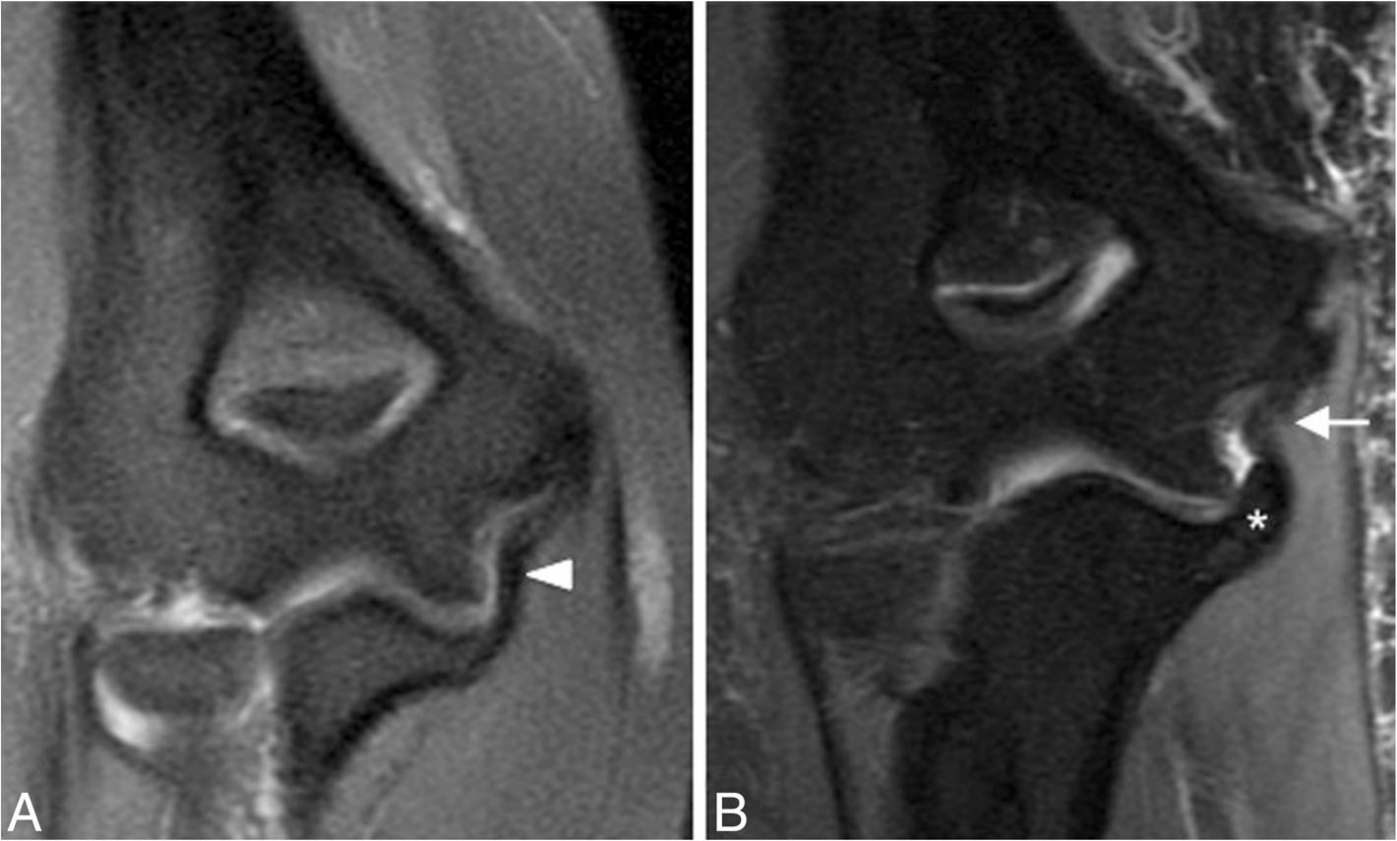
Two different patients with chronic elbow pain and medial instability. Coronal FS PD-weighted MRI (**a**) showing a chronically thickened anterior bundle of the medial collateral ligament (white arrowhead). Coronal FS PD-weighted MRI (**b**) showing a chronically thickened anterior bundle of the medial collateral ligament (white arrow), and a deformity of the sublime tubercle due to malunion of an old fracture (white asterisk)

**Fig. 19:**
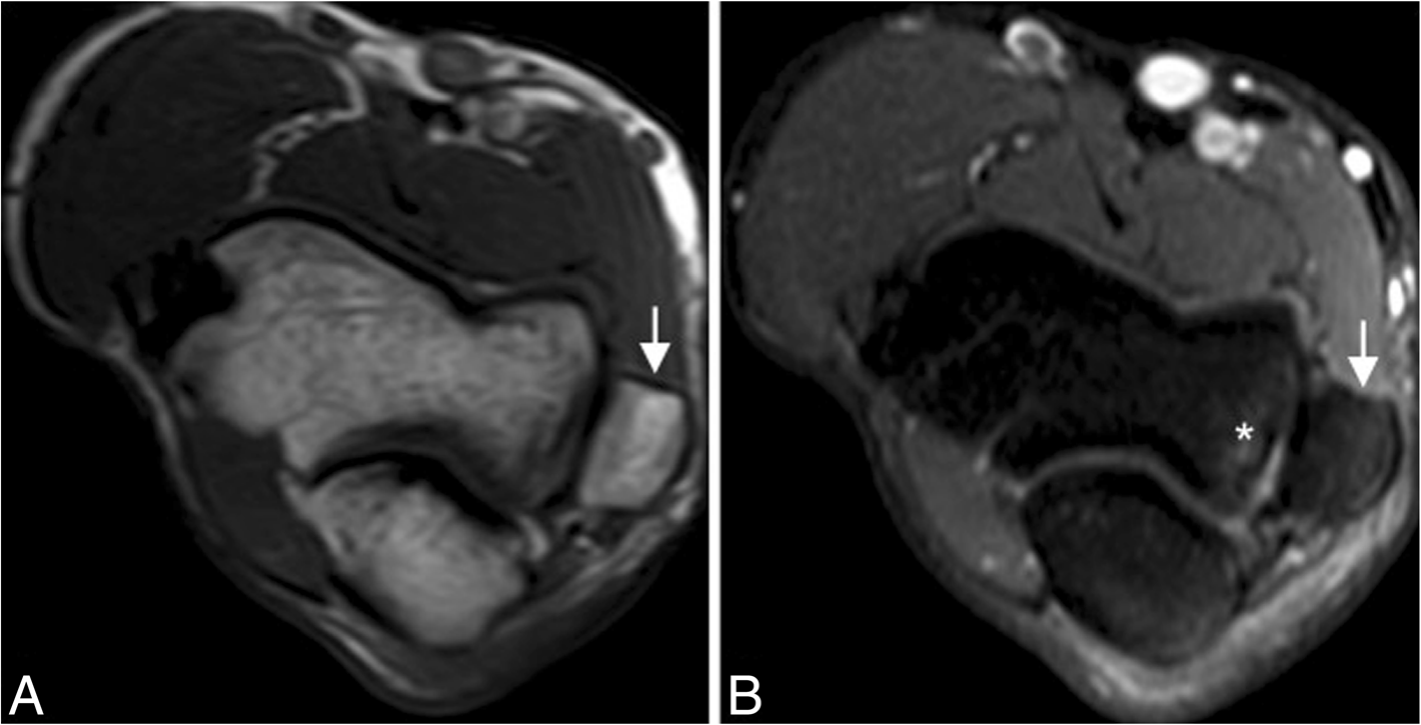
A 50-year-old man with elbow pain and decreased range of motion due to an apophyseal injury sustained before physeal closure. Axial T1-weighted MRI (**a**) and axial FS PD-weighted MRI (**b**) showing chronic non-union fracture of the medial epicondyle and heterotopic ossification along the anterior band of the medial collateral ligament (white arrows). Note stress reaction, manifest as bone marrow edema (white asterisk)

In chronic cases, MRI may show thickening, abnormal signal, and discontinuity of the ligament (Fig. 18). The development of heterotopic ossification along the course of the ligament has been described (Fig. 19).

**Fig. 20:**
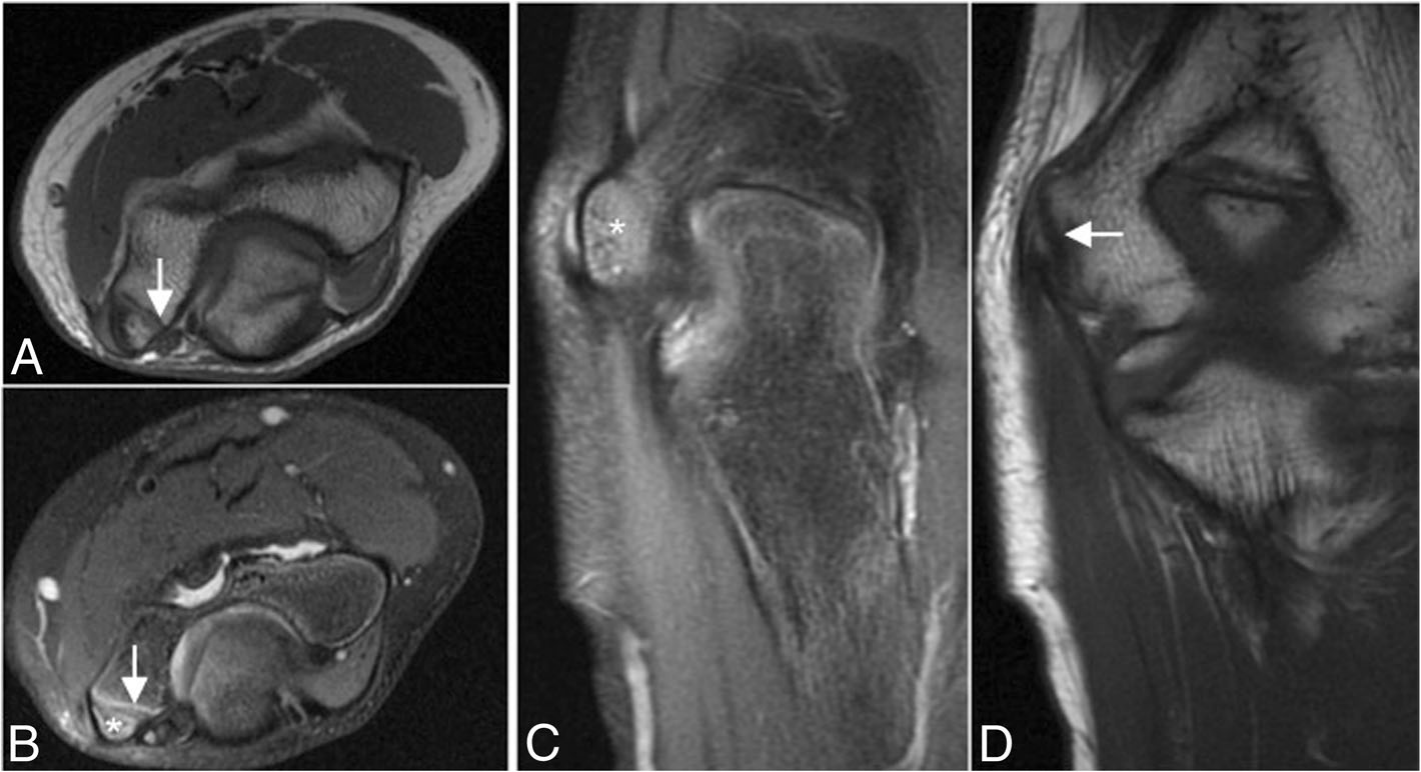
A 12-year-old left-handed baseball pitcher with medial epicondyle pain. Axial T1-weighted MRI (**a**), axial FS PD-weighted MRI (**b**), coronal FS PD-weighted MRI (**c**), and coronal T1-weighted MRI (**d**) showing a widening of the medial epicondylar physis (white arrows) and bone marrow edema (white asterisks)

In skeletally immature individuals, a chronic valgus stress causes a repetitive traction on the apophysis by the common flexor tendon and the MCL, causing a medial epicondyle apophysitis, also known as Little leaguer’s elbow [5, 30–32]. Sometimes, the epicondyle avulses into the joint and can simulate an osseous body. More often, it remains close to the parent bone, presenting on MRI with bone marrow edema and/or a widened gap between the medial epicondyle and the humerus (Fig. 20). Non-union can lead to repeated valgus instability.

### Lateral collateral ligament complex injury

The LCL complex resists excessive varus and external rotational stress. Varus stress applied to the elbow may be due to an acute injury, but rarely to repetitive stress, as encountered on the medial side. Tears can involve one or more of the three bundles, but the LUCL is the most important in terms of stability [31]. However, kinematic studies refer to both the LUCL and RCL working in concert to resist valgus stress. If both are injured, it can secondarily lead to subluxation or dislocation of the radiocapitellar joint even with an intact annular ligament, usually in the setting of chronic or repeated injury. LUCL tears usually involve the humeral origin [2, 5]. Failure to recognize LCL complex tears prior to surgical treatment of tennis elbow, particularly the LUCL, will lead to persistent postoperative symptoms.

Injuries of the LCL complex can occur in patients with advanced cases of tennis elbow, who also have tears of the common extensor tendon, and after a fall on the outstretched hand. Among iatrogenic causes of LCL complex disruption, we find overaggressive extensor tendon release for lateral epicondylitis, and radial head excision for comminuted fractures of the radial head [33, 34]. Repeated corticosteroid injections into the common extensor tendon and LCL complex origins might contribute to the weakening and ultimate failure of these structures [34].

**Fig. 21:**
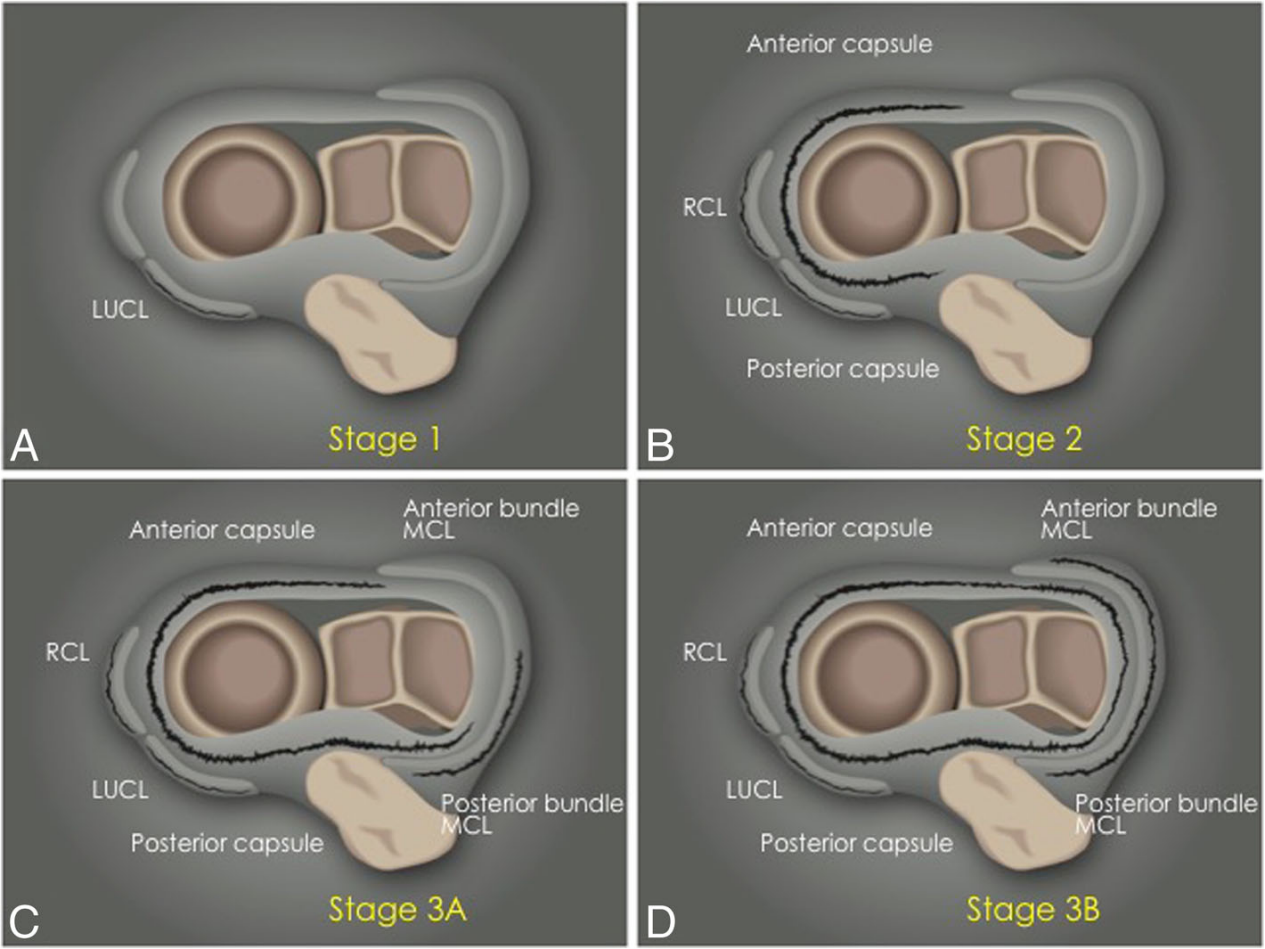
Axial illustrations with superior view demonstrating the progression of the structures injured in the posterolateral rotatory instability

**Fig. 22:**
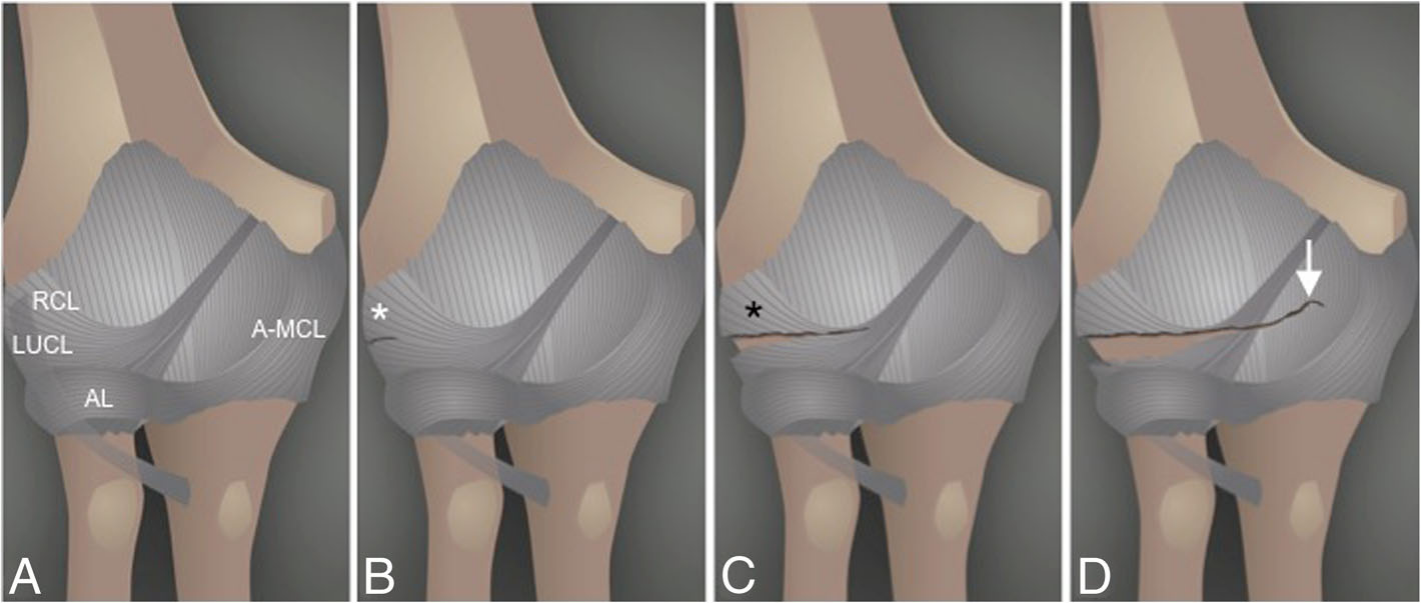
Anterior view of the elbow demonstrating the progression of the structures injured in a posterior dislocation, from lateral to medial. **a** Normal. **b** Proximal disruption of the lateral ulnar collateral ligament (white asterisk). **c** Proximal disruption of the lateral ulnar collateral ligament and the radial collateral ligament (black asterisk). **d** Anterior capsule tear (white arrow). Lateral ulnar collateral ligament (LUCL). Radial collateral ligament (RCL). Annular ligament (AL). Anterior bundle of the medial collateral ligament complex (A-MCL)

The most common pattern of recurrent elbow instability is posterolateral rotatory instability (PLRI) [7]. It represents a spectrum of pathology consisting of three stages depending on the grade of soft-tissue disruption, which extends from lateral to medial, the so-called circle of Hori [5, 7, 17] (Figs. 21 and 22).

**Fig. 23:**
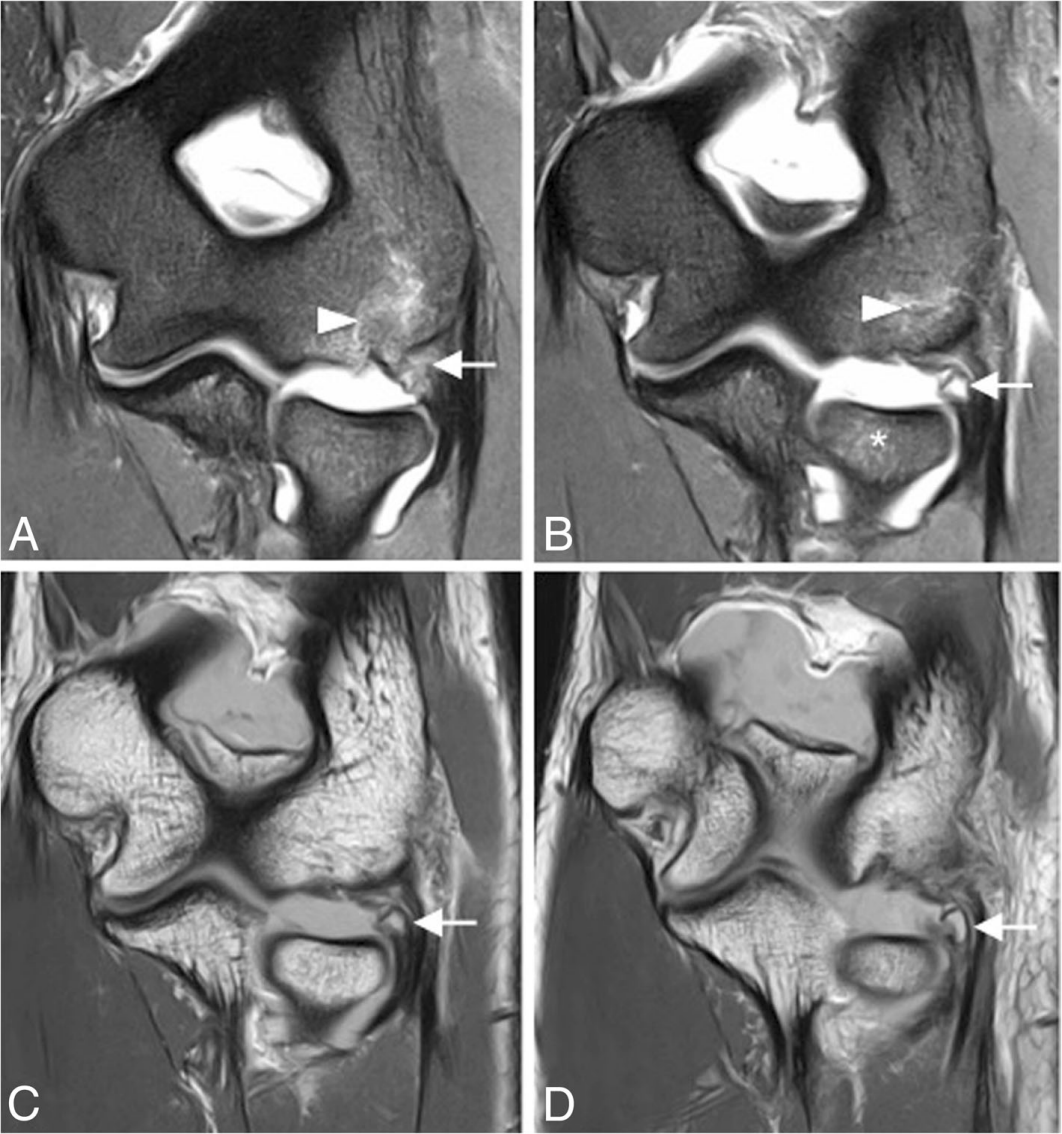
Posterolateral rotatory instability, stage 1. Coronal FS PD-weighted MRI (**a**, **b**) and coronal T1-weighted MRI (**c**, **d**) showing an avulsion of the proximal lateral ulnar collateral ligament and retraction of the osseous fragment (white arrows). Bone marrow edema is seen in the capitellum (white arrowheads) and radial head (white asterisks)

**Fig. 24:**
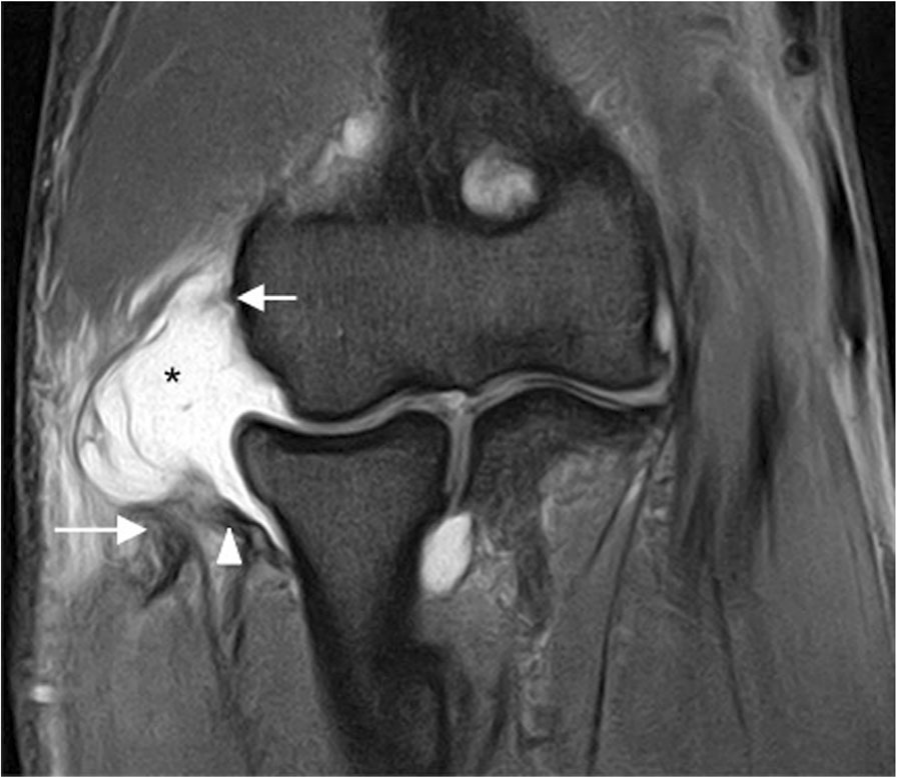
Posterolateral rotatory instability, stage 2. Coronal FS PD-weighted MRI showing a complete detachment of the proximal lateral ulnar collateral ligament, the proximal radial collateral ligament proper, and the common extensor tendon (white short arrow), with retraction of the lateral ulnar collateral ligament (white arrowhead) and common extensor tendon (long white arrow). Note the extravasation of the joint fluid through the tear (black asterisk)

**Fig. 25:**
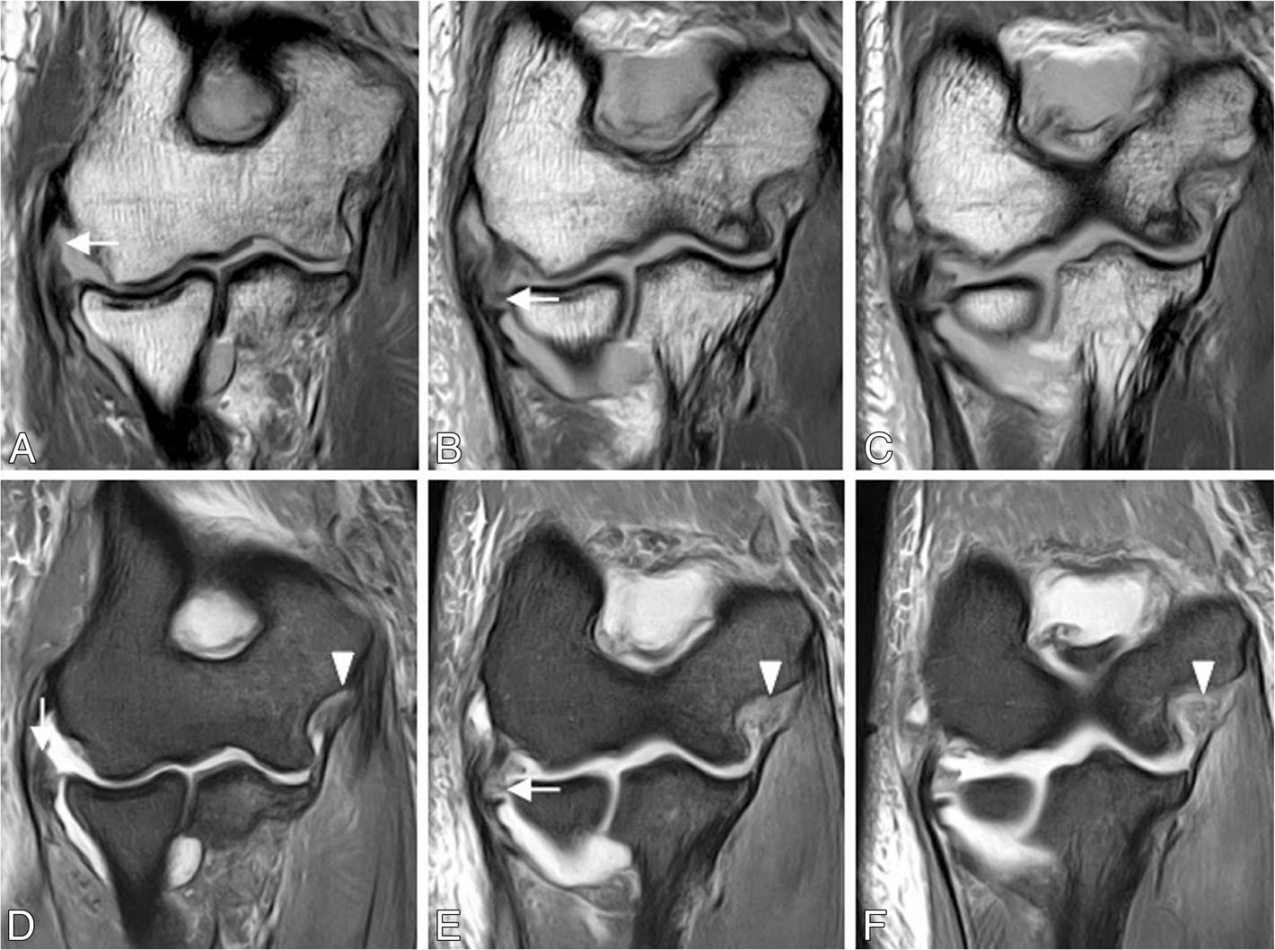
Posterolateral rotatory instability, stage 3B. Consecutive coronal T1-weighted MRI (**a**–**c**), and consecutive coronal FS PD-weighted MRI (**d**–**f**) showing an acute proximal full-thickness tear of the lateral ulnar collateral ligament and radial collateral ligament (white arrows) and anterior bundle of the medial collateral ligament complex sprain (white arrowheads)

**Fig. 26:**
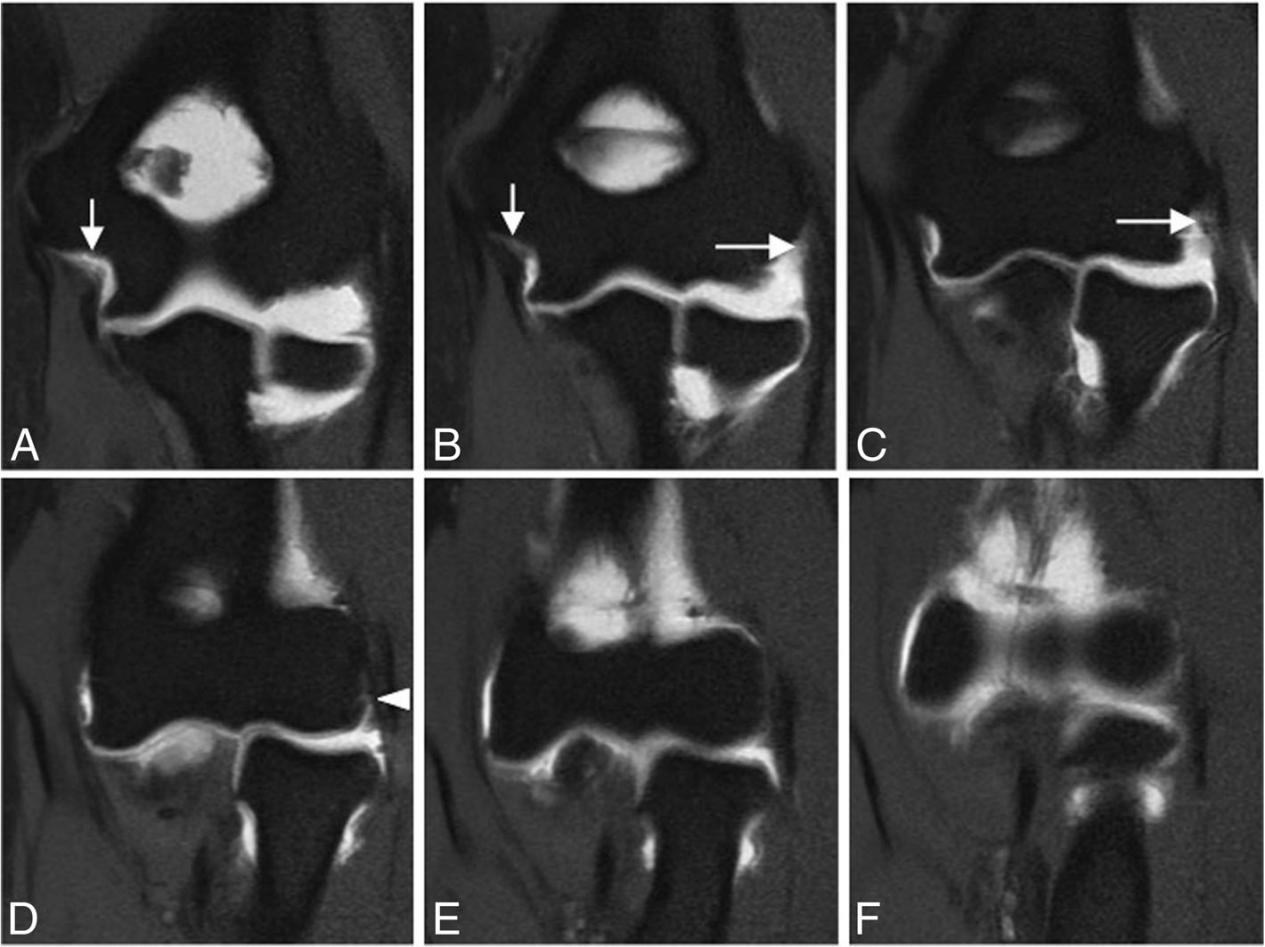
Posterolateral rotatory instability, stage 3B. Consecutive coronal FS T1-weighted direct MR arthrographic images (**a**–**f**) showing a proximal partial-thickness tear of the anterior bundle of the medial collateral ligament complex (short arrows), proximal complete tear of the lateral ulnar collateral (long white arrows), and partial-thickness tear of the radial collateral ligament proper (white arrowhead)

**Fig. 27:**
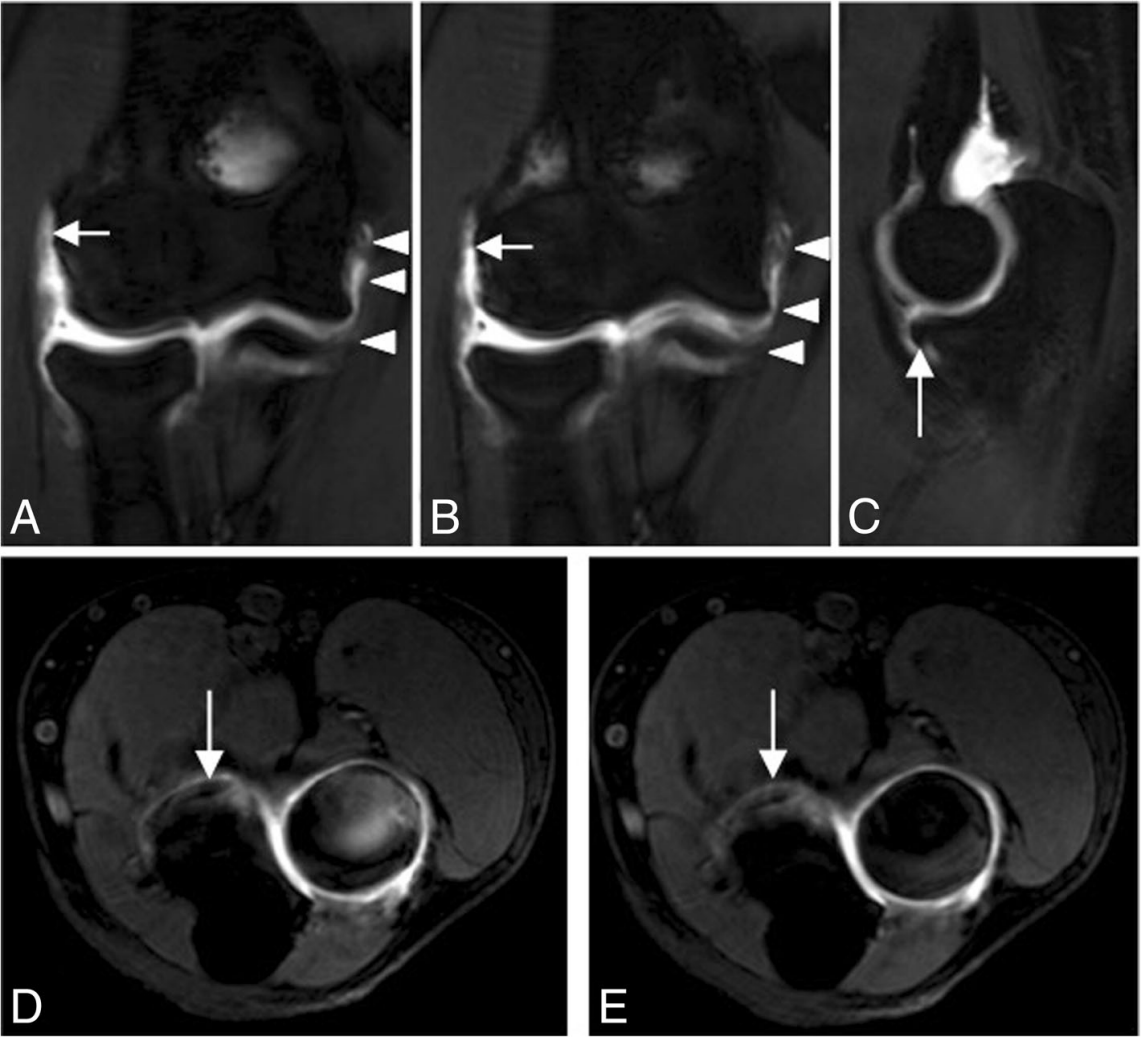
Posterolateral rotatory instability, stage 3B. Consecutive coronal FS T1-weighted direct MR arthrographic images (**a**, **b**), sagittal FS T1-weighted direct MR arthrographic image (**c**), and consecutive axial FS T1-weighted direct MR arthrographic images (**d**, **e**) showing a proximal complete tear of the lateral ulnar collateral ligament and radial collateral ligament proper (white short arrows), diffuse partial-thickness tear of the anterior bundle of the medial collateral ligament complex (white arrowheads), and a non-displaced fracture of the coronoid process (long white arrows)

**Fig. 28:**
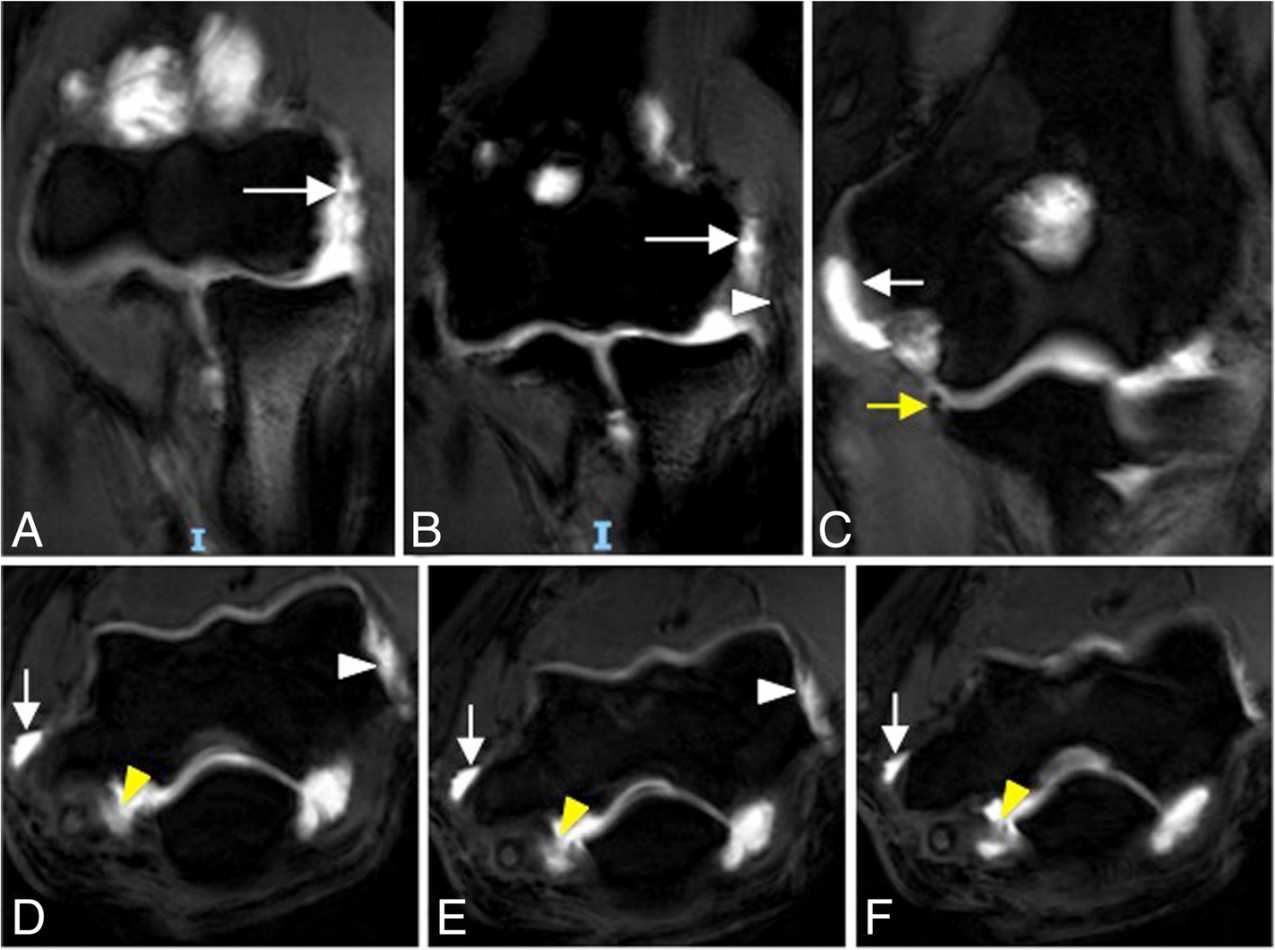
Posterolateral rotatory instability, stage 3C. Consecutive coronal FS T1-weighted direct MR arthrographic images (**a**–**c**), and consecutive axial FS T1-weighted direct MR arthrographic images (**d**–**f**) demonstrating a full-thickness tear of the anterior bundle of the medial collateral ligament complex (yellow arrow), proximal full-thickness tear of the common flexor tendon (short white arrows), proximal full-thickness tear of the common extensor tendon (white arrowheads), proximal complete tear of the lateral ulnar collateral ligament and radial collateral ligament proper (long white arrows), and a complete tear of the posterior bundle of the medial collateral ligament complex (yellow arrowheads)

- In stage 1, there is posterolateral subluxation of the ulna on the humerus, which results in insufficiency or tearing of the LUCL (Fig. 23).
- In stage 2, the elbow dislocates incompletely, so that the coronoid process is perched under the trochlea. The RCL, and the anterior and posterior articular capsule are disrupted, in addition to the LUCL (Fig. 24).
- In stage 3, the elbow dislocates completely with progressive disruption of the MCL and the coronoid process rests behind the humerus. The LUCL, the RCL, and the articular capsule are disrupted. Stage 3 is further subdivided into three categories:
  - **◦** Stage 3A: disruption of the posterior bundle of the MCL while the anterior bundle of the MCL remains intact.
  - **◦** Stage 3B: disruption of both the posterior and anterior bundle of the MCL. (Figs. 25, 26, and 27).
  - **◦** Stage 3C: the entire distal humerus is stripped off soft tissues, rendering the elbow grossly unstable even when a splint or cast is applied with the elbow in a semi-flexion position (Fig. 28).

The classic clinical presentation of patients with PLRI includes pain as well as a sensation of locking, clicking, or snapping when the arm moves from a flexed to an extended elbow position. Ultimately, the diagnosis of PLRI is based on history and physical examination using provocative maneuvers. The *pivot shift test of the elbow* is designed to test for PLRI due to insufficiency of the LUCL and the RCL [2]. Diagnosis is often difficult, as the clinical exam can be misleading unless performed under anesthesia. MR imaging can therefore be extremely useful in evaluating for tears of the LUCL in patients presenting with lateral elbow pain or instability. It is best evaluated in coronal oblique, coronal, and axial planes. Associated posterolateral subluxation of the radial head is best appreciated on sagittal images [5]. LUCL tears may appear as an isolated finding in patients with PLRI in stage 1, or they can be detected in association with the rupture of the MCL in stage 3B. MR imaging is also useful in assessment of the cubital tunnel retinaculum, and the ulnar nerve in posterior dislocations.

**Fig. 29:**
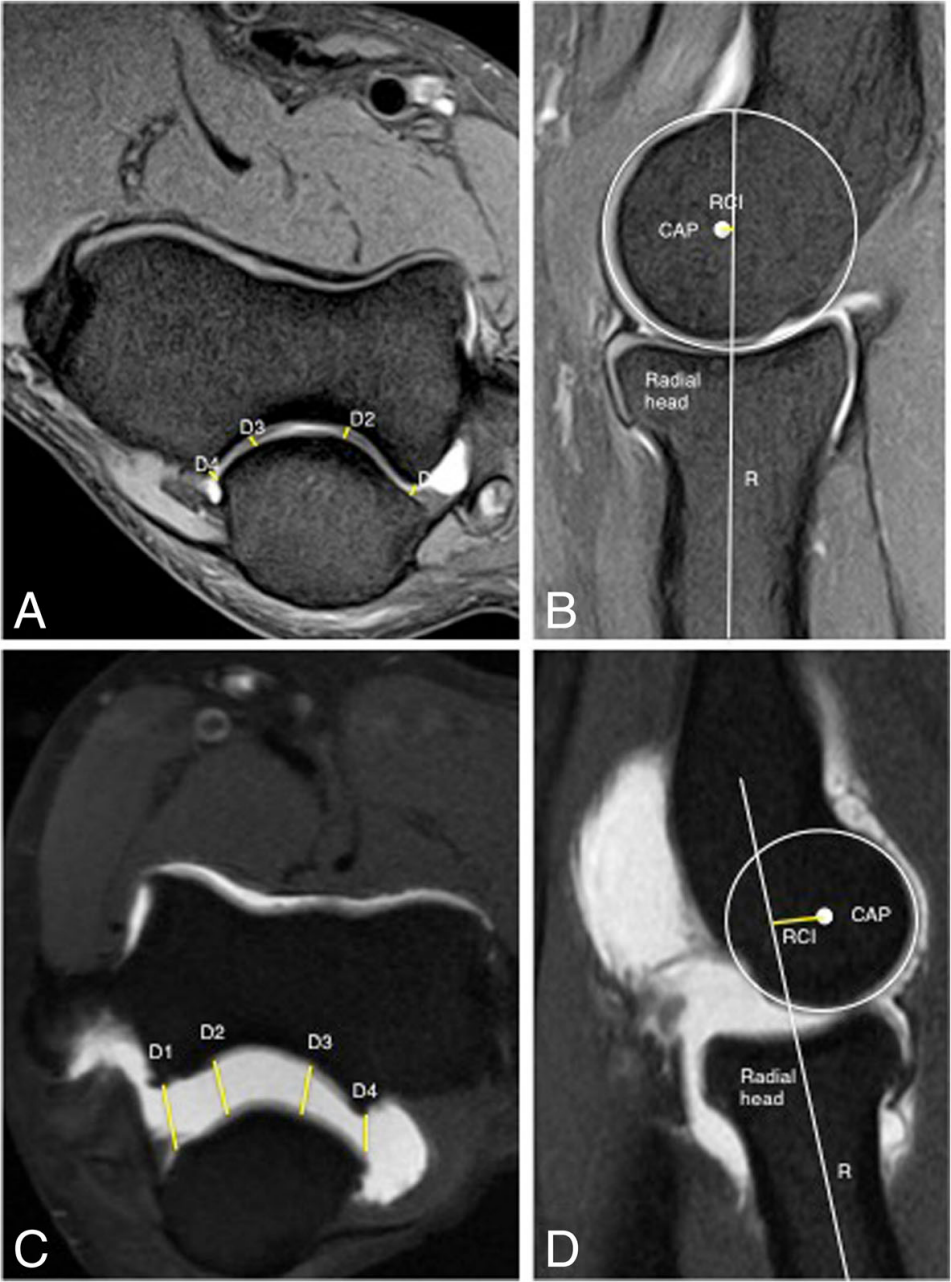
Axial FS PD-weighted MRI (**a**) showing the measurement of normal ulnohumeral incongruity: the distance between the trochlear joint surface and olecranon joint surface (D1, D2, D3, and D4). Sagittal FS PD-weighted MRI (**b**) showing the measurement of normal radiocapitellar incongruity (RCI): the distance between CAP and R. Axial FS PD-weighted MRI showing an ulnohumeral incongruity of more 1 mm (**c**). Sagittal FS PD-weight MRI showing a radiocapitellar incongruity of more 2 mm (**d**)

Incongruity of the elbow can be considered as an indirect sign of instability and can be evaluated with MRI. There is a significant increase in joint incongruity in unstable elbows analyzed in sagittal view through the radial head and in axial view through the motion axis of the distal humerus compared with stable elbow joints. In a sagittal view through the center of the radial head, the radiocapitellar incongruity is the distance between the rotational center of the capitellum (CAP) and a line along the longitudinal axis of the radius through the center of the radial head (R) (Fig. 29b). In an axial view through the motion axis of the distal humerus, the ulnohumeral incongruity is the difference of the lowest and the highest values of four measures extending from the trochlear joint surface to the corresponding joint surface of the olecranon (Fig. 29a) [35]. Posterolateral translation of the radial head of more than 1.2 mm and axial ulnohumeral incongruity of more than 0.7 mm are cutoffs that can be used as screening tools to aid diagnosis of elbow instability [5, 35]. Radiocapitellar incongruity of more than 2 mm (Fig. 29d) and axial ulnohumeral incongruity of more than 1 mm are highly suspicious of elbow instability (Fig. 29c). To exclude the diagnosis of instability, radiocapitellar and axial ulnohumeral incongruity have to be below 0.3 mm [35].

**Fig. 30:**
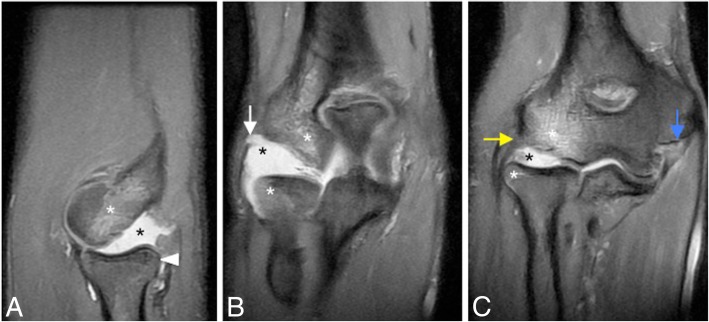
Posterolateral rotatory instability, stage 3B. Sagittal FS PD-weighted MRI (**a**) and consecutive coronal FS PD-weighted MRI (**b**, **c**) showing a posterolateral subluxation of the radial head (white arrowhead), proximal disruption of the lateral ulnar collateral ligament (white arrow), partial proximal disruption of the radial collateral ligament (yellow arrow), proximal disruption of the anterior bundle of the medial collateral ligament (blue arrow), radial head and posterior capitellar contusions (white asterisks), and joint effusion (black asterisks)

**Fig. 31:**
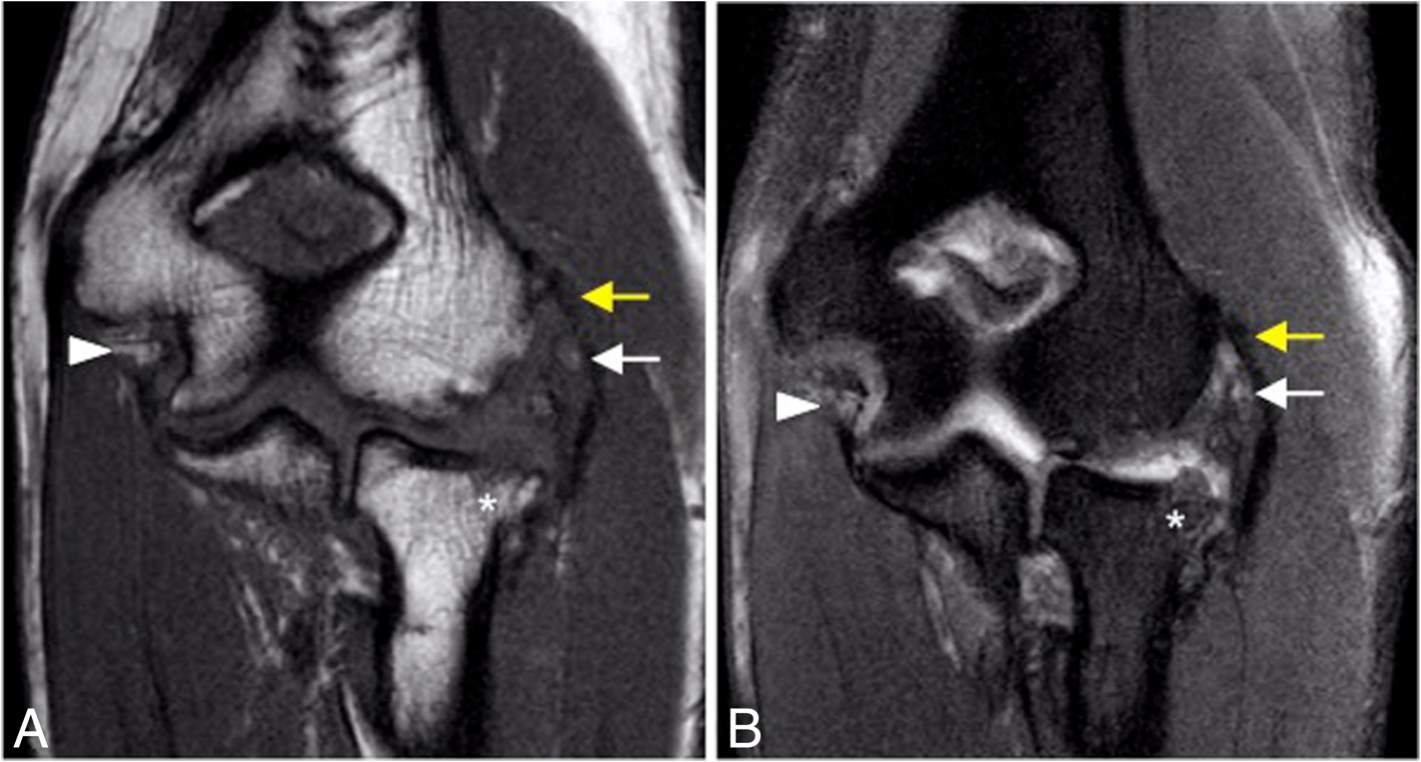
Posterolateral rotatory instability, stage 3B. Coronal T1-weighted MRI (**a**) and coronal FS PD-weighted MRI (**b**) showing an acute proximal common avulsion of the lateral ulnar collateral ligament and radial collateral ligament (white arrows), an acute avulsion of the anterior bundle of the medial collateral ligament complex (white arrowheads), a radial head fracture (white asterisks), a partial tear of the proximal common extensor tendon (yellow arrows), and joint effusion

**Fig. 32:**
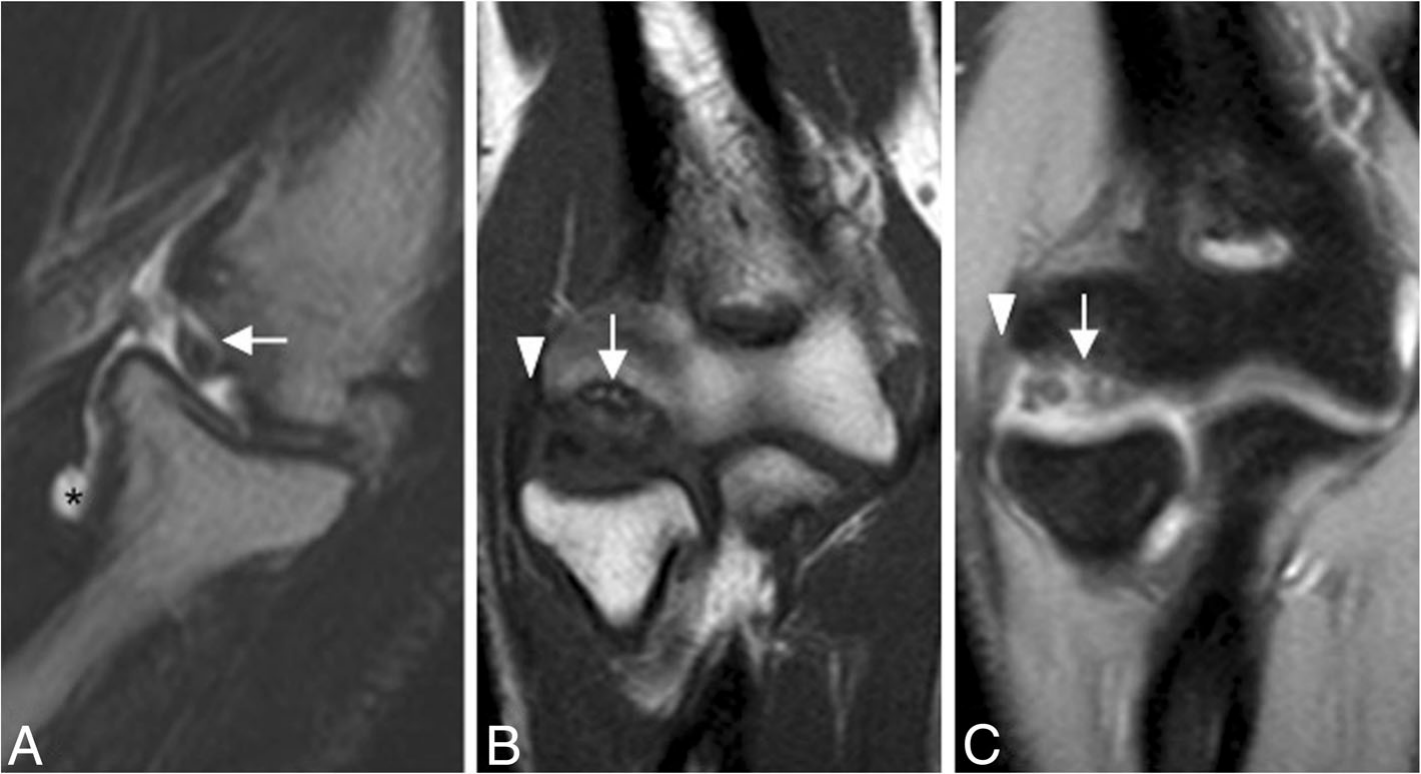
A 33-year-old female gymnast with elbow pain and instability. Sagittal T2-weighted MRI (**a**), coronal T1-weighted MRI (**b**), and coronal FS PD-weighted MRI (**c**), demonstrating an osteochondral lesion in the posterior capitellum caused by posterolateral rotatory instability. The lesion is unstable, with fluid extending into the interface between the fragment and the parent bone (white arrows). Proximal partial disruption of the lateral ulnar collateral ligament (white arrowheads). Joint effusion (black asterisk)

MR imaging findings in acute LUCL injury resemble those described for the MCL: hyperintensity, discontinuity, and surrounding soft-tissue edema on conventional fluid-sensitive MR images (Figs. 23, 24, and 25). Osteochondral lesions and bone contusions may be seen at the posterolateral margin of the capitellum as well as at the radial head, and coronoid process (Figs. 30 and 31). The role of imaging is to provide information regarding the integrity of the overlying articular cartilage, the viability and stability of the separated fragment (Fig. 32), and the presence of associated intra-articular bodies.

The appearance of chronically torn and remodeled LUCL is similar to that described for the MCL, with thickening, abnormally increased signal, and discontinuity as possible findings (Figs. 26, 27, and 28).

**Fig. 33:**
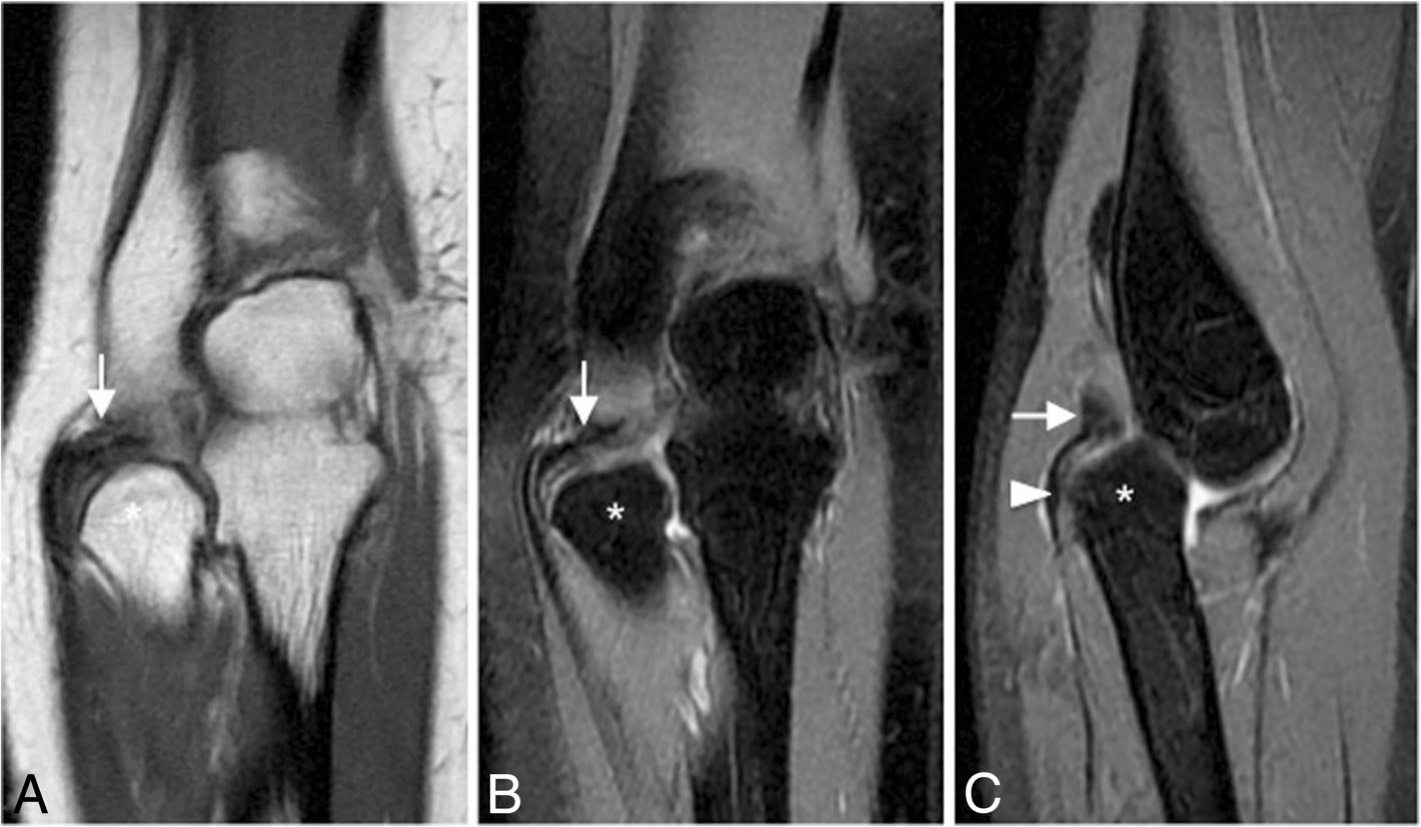
A 40-year-old man with recurrent painful click in the elbow. Coronal T1-weighted MRI (**a**), coronal FS PD-weighted MRI (**b**), and sagittal FS PD-weighted MRI (**c**) showing a chronic rupture and displacement of the annular ligament (white arrows), dislocation of the radial head out of the annular ligament sling (white arrowhead), and radial head deformity (white asterisks)

**Fig. 34:**
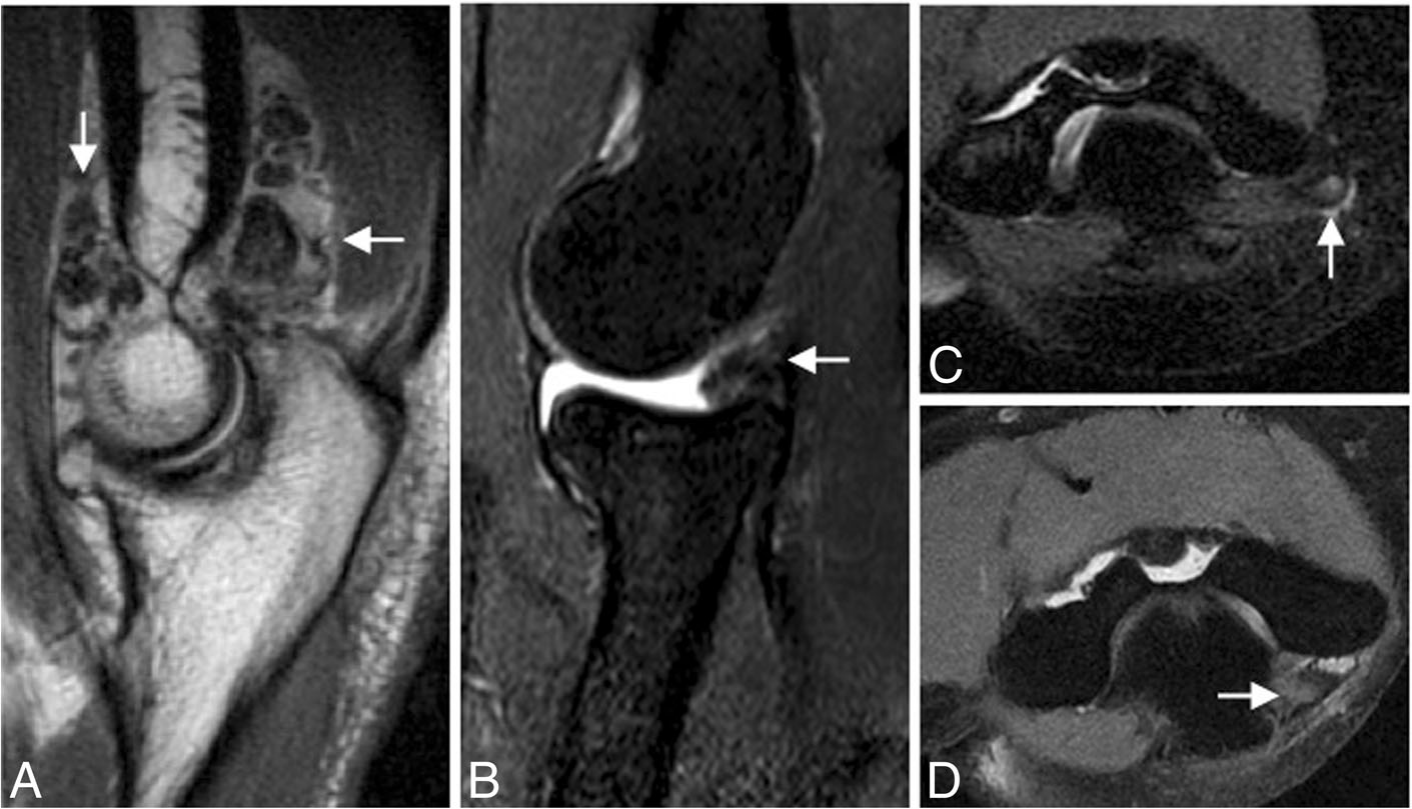
Four different patients with recurrent painful click in the elbow. Sagittal T2-weighted MRI (**a**), sagittal FS PD-weighted MRI (**b**), axial FS PD-weighted MRI (**c**), and axial T1-weighted MRI (**d**) showing synovial osteochondromatosis (**a**), a posterolateral plica (**b**), an ulnar nerve subluxation (**c**), and hypertrophy of the medial head triceps (**d**)

The AL can be injured in the setting of trauma and after closed reduction of elbow dislocation (Figs. 2 and 33). A tear of one or more components of the LCL complex is often associated with intra-articular displacement of the annular ligament. In adults, sole injury to the AL is rare. Most often, it is associated with a larger injury to the LUCL complex, including varus elbow stress, elbow dislocation, and PLRI. However, in pediatric population, dislocation and rupture of the AL can be seen in the setting of nursemaid elbow and Monteggia fracture [36]. The AL is best visualized on axial and sagittal MR imaging using standard sequences and field strength. The clinical feature of posterior dislocation and chronic AL injury may be a recurrent painful “click.” Other differential diagnoses are intra-articular loose bodies, a posterolateral plica, an ulnar nerve subluxation, and a snapping triceps syndrome (Fig. 34).

Elbow fractures with ulnohumeral instability tend to occur in five general patterns: radial head fracture with ulnohumeral dislocation, terrible triad, varus posteromedial rotatory instability (VPMRI), olecranon fracture dislocation (OFD), and lateral column fracture of the distal humerus with ulnohumeral dislocation. VPMRI consists of a fracture of the anteromedial coronoid facet and a rupture of the LCL complex. OFD consists of a fracture of the olecranon with subluxation/dislocation of the intact forearm relative to the distal humerus. This is usually accompanied by a radial head fracture. A “terrible triad” consists of a posterior elbow dislocation, radial head fracture, coronoid process fracture, and a rupture of the LCL complex. These patients are at high risk for chronic instability [37].

Displaced radial head and neck (DRHN) fracture is always a complex fracture caused by the combination of a valgus force and pathologic forearm external rotation. They are accompanied by collateral ligament injuries and bony contusion. According to the Charalambous classification [38], type 3D and 4D DRHN fractures tended to have a higher association with MCL rupture compared with type 1D and 2D DRHN fractures, commonly associated with LUCL rupture, although this was not statistically significant [39].

Another important consideration with respect to elbow dislocation is that, as the ring of soft tissues is disrupted posterolaterally to medially, the articular capsule is torn and insufficient. Therefore, fluid in the elbow joint can escape through the capsular tear and a joint effusion, which is an indirect sign of elbow trauma, may not be present.

### Ligaments: postoperative

**Fig. 35:**
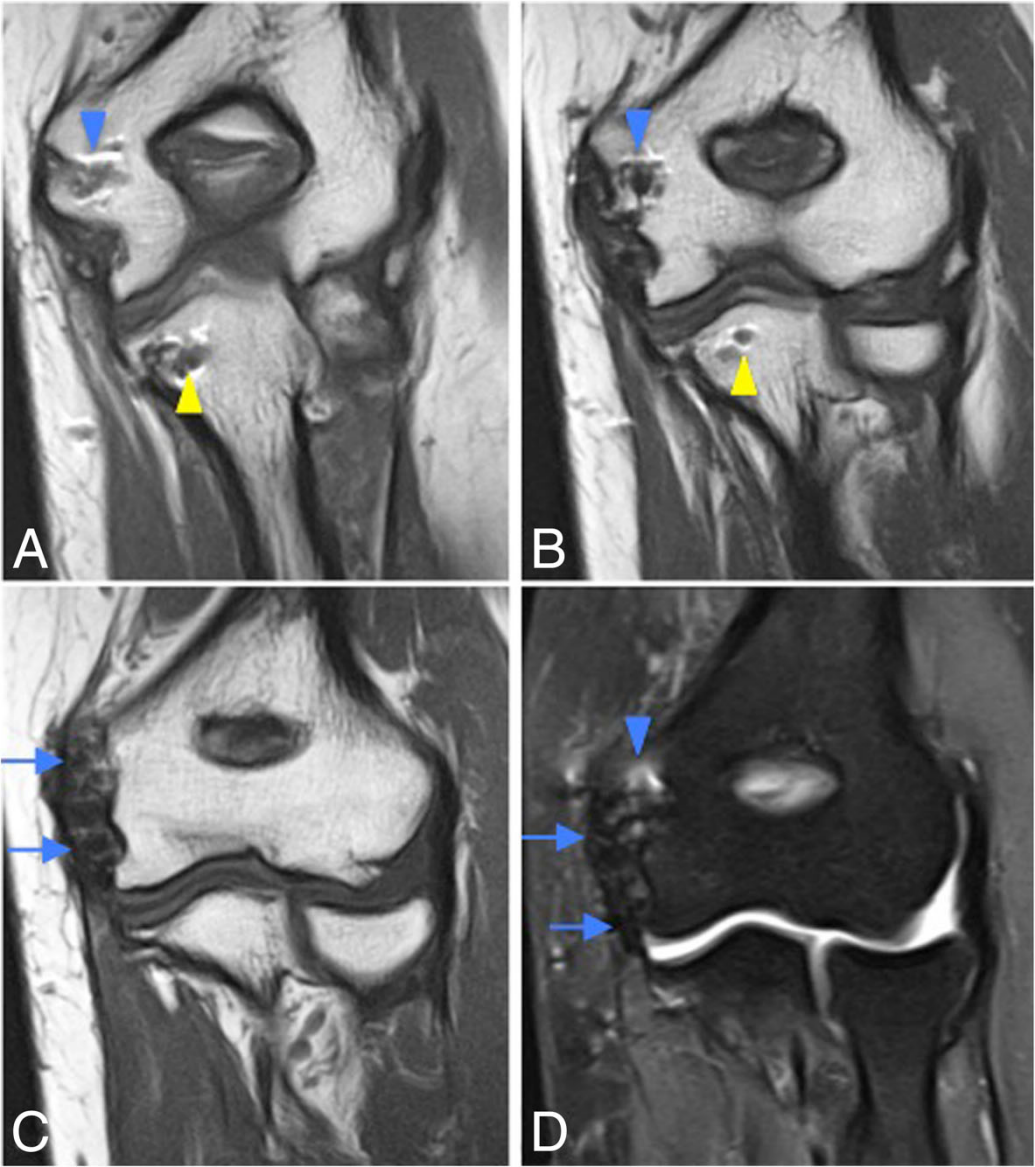
Normal appearance of reconstructed medial collateral ligament (docking technique). Consecutive coronal T1-weighted MRI (**a**–**c**) and coronal FS PD-weighted MRI (**d**). The intact graft (blue arrows) extends from humeral tunnel (blue arrowheads) to ulnar tunnel (yellow arrowheads)

**Fig. 36:**
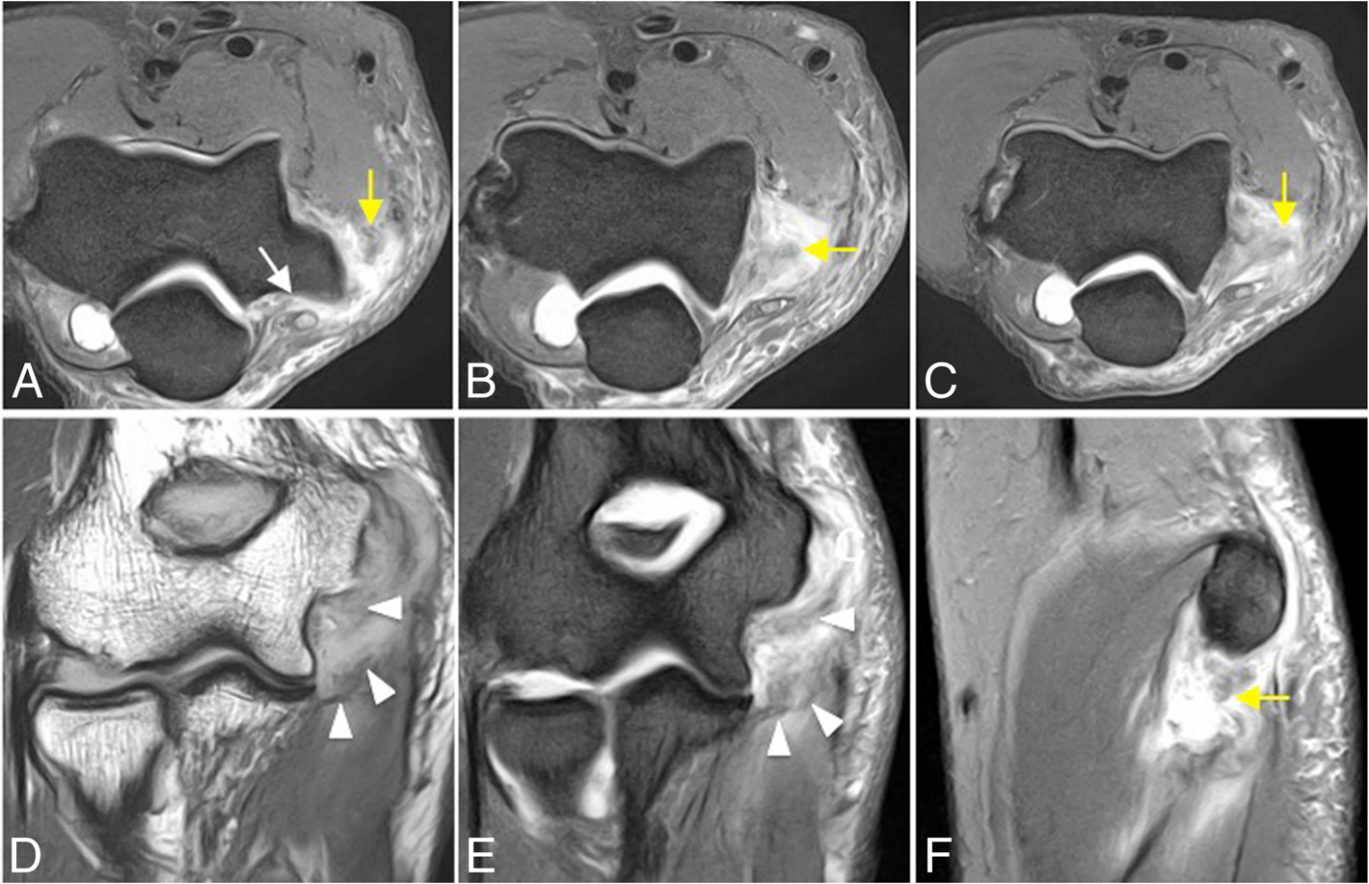
Acute subluxation in a handball player with a history of medial instability treated with arthroscopic repair. Consecutive axial FS PD-weighted MRI (**a**–**c**), coronal T1-weighted MRI (**d**), coronal FS PD-weighted (**e**), and sagittal FS PD-weighted MRI (**f**) showing a re-tear of the repaired anterior bundle of the medial collateral ligament (white arrowheads), a complete tear of the common flexor tendon (yellow arrows), and a tear of the posterior bundle of the medial collateral ligament (white arrow)

It is important to recognize that intact postoperative ulnar and lateral collateral ligaments may be thicker and more heterogeneous in signal intensity than native ligaments (Fig. 35). However, retears can be detected by the same criteria of partial or complete ligamentous discontinuity (Fig. 36). Anchoring materials can cause imaging artifacts, although it is usually not necessary to modify the conventional MRI protocol [40].

**Fig. 37:**
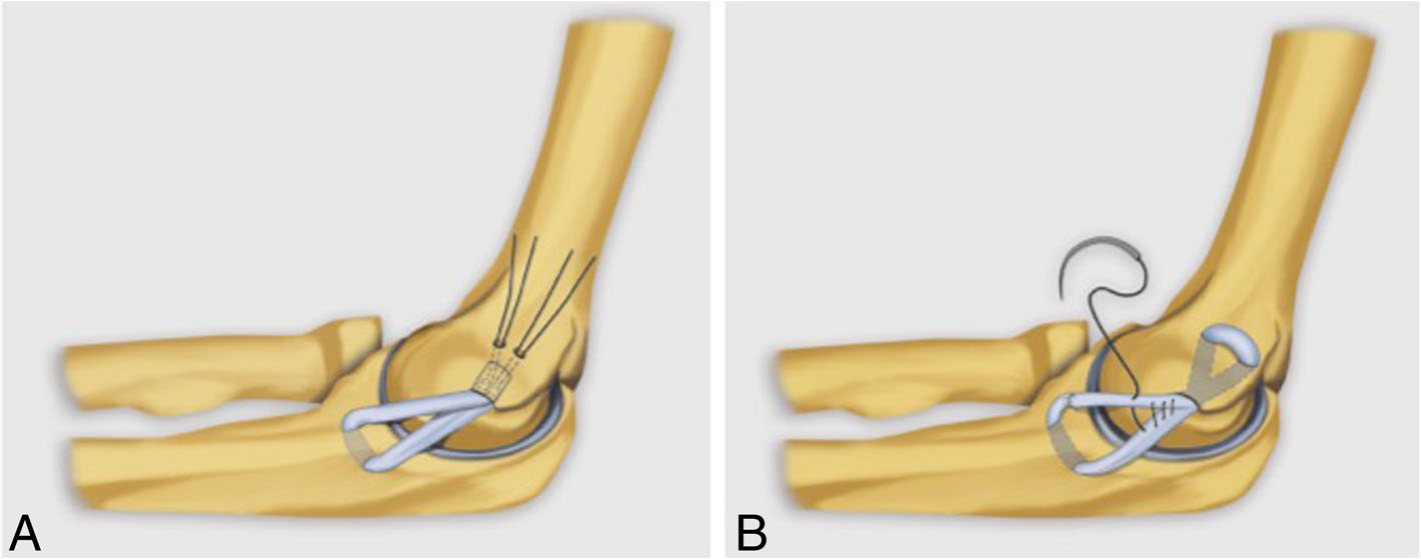
Elbow illustrations demonstrating docking technique (**a**) and Jobe technique (**b**) for medial collateral ligament reconstruction

On the medial side, surgery may be indicated in high-level athletes and manual workers with persistent symptoms of instability and elbow pain after 6 months of adequate conservative treatment. Primary repair techniques generally yield poor results (Fig. 36), and ligament reconstruction with tendinous graft is preferred. There are several techniques for MCL complex reconstruction. The main reconstructive techniques of the MCL complex include the modified Jobe technique, the docking technique, and the interferential screw technique. These techniques use a free graft harvested from the palmaris longus or semitendinosus tendons, which is placed through one or more osseous tunnels in the humerus and ulna oriented in a manner that simulates the anatomy of the A-MCL. The Jobe technique (Fig. 37b) involves the dissection of the origin of the flexor-pronator muscles and transposition of the ulnar nerve. The main complication of this technique is secondary ulnar neuropathy. The modified Jobe technique involves a longitudinal incision of the flexor carpi ulnaris, which reduces the incidence of ulnar neuropathy. The docking technique (Figs. 35 and 37a) is a variant of the modified Jobe technique, with simplification of the humeral bone tunnels [40, 41].

**Fig. 38:**
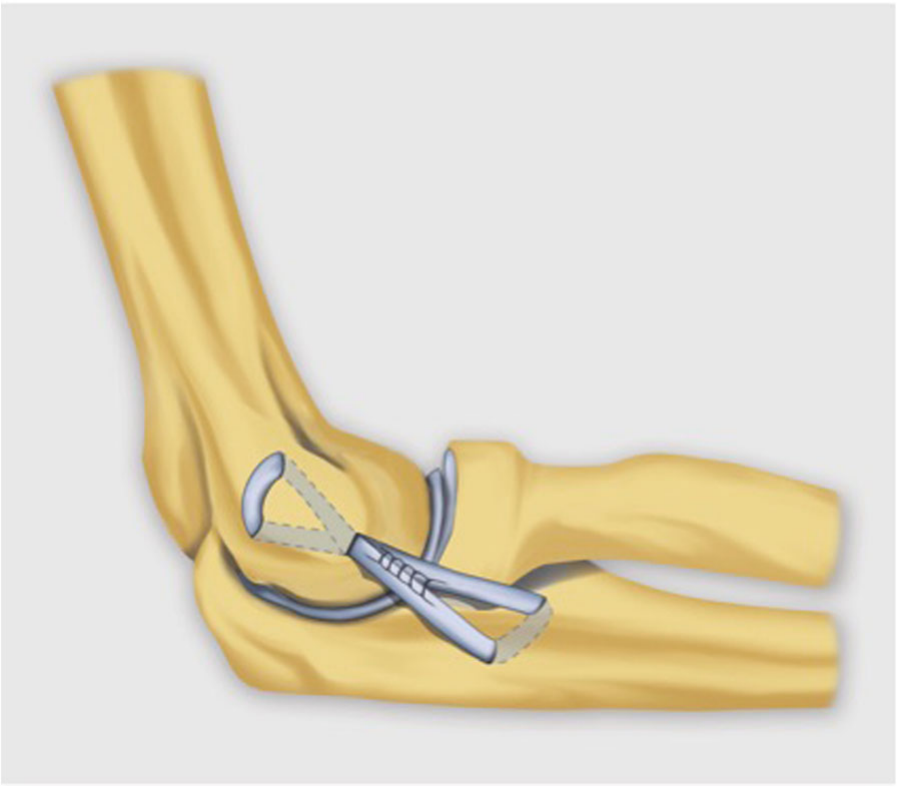
Lateral view of the elbow demonstrating the open reconstruction technique for lateral ulnar collateral ligament reconstruction

Posterolateral rotatory instability often requires surgical treatment. The LUCL is reconstructed with a free tendon graft, which is fixated to one or more osseous tunnels in the lateral humeral epicondyle proximally and the sublime tubercle of the ulna distally in an orientation that simulates its anatomy (Fig. 38) [40, 42].

## Summary

Conventional MRI and MR arthrography are the imaging modalities of choice in the evaluation of elbow ligament injuries. Proper coil selection, pulse sequence parameters, and patient positioning enhance the ability of MR imaging to demonstrate subtle injuries to the ligament and the regional osseous and soft-tissue structures. Anatomical and biomechanical knowledge of the support structures that provide stability to the medial and lateral elbow is essential to correctly interpret the pathological findings. Familiarity with the associated injuries that can be seen in MCL and LCL complex ruptures will therefore improve detection of ligament abnormalities. MR imaging is useful in the evaluation of children with elbow pain, as it can demonstrate physeal as well as ligamentous and osseous injury.
